# The lupus damage index revision program: Results from the item generation and reduction phases

**DOI:** 10.1136/lupus-2026-002282

**Published:** 2026-08-12

**Authors:** Burak Kundakci, Megan R W Barber, Ann E Clarke, Sindhu R Johnson, Anselm Mak, Hermine I Brunner, Murat Inanc, Rosalind Ramsey-Goldman, Guillermo Ruiz-Irastorza, Ellen M Ginzler, John G Hanly, Alexandra Legge, Cynthia Aranow, Laurent Arnaud, Nathalie Costedoat-Chalumeau, Dafna D Gladman, Søren Jacobsen, Michelle A Petri, Clovis A Silva, Evelyne Vinet, Alexandre E Voskuyl, SDI Revision Leadership Team, Ian N. Bruce, SDI Revision Literature Review Groups, Jiacai Cho, SDI Revision Delphi Panel, Alfredo Aguirre, SDI Revision Item Reduction Committee, Laurent Arnaud, SDI Revision Clinical Domain Groups, Sulaiman Al-Mayouf, Ian N Bruce

**Affiliations:** 1 Centre for Musculoskeletal Research, Division of Musculoskeletal and Dermatological Sciences, Faculty of Biology, Medicine and Health, The University of Manchester, Manchester, England, UK; 2 School of Medicine and Population Health, The University of Sheffield, Sheffield, England, UK; 3 Division of Rheumatology, Cumming School of Medicine, University of Calgary, Calgary, Alberta, Canada; 4 Institute of Health Policy, Management and Evaluation, University of Toronto, Toronto, Ontario, Canada; 5 Division of Rheumatology, Department of Medicine, Schroeder Arthritis Institute, Krembil Research Institute, Toronto Western and Mount Sinai Hospitals, Toronto, Ontario, Canada; 6 Division of Rheumatology & Allergy, Department of Medicine, Yong Loo Lin School of Medicine, National University of Singapore, Singapore; 7 Department of Pediatrics and Cincinnati Children’s Hospital Medical Center, University of Cincinnati, Cincinnati, Ohio, USA; 8 Division of Rheumatology, Department of Internal Medicine, Istanbul Medical Faculty, Istanbul University, Fatih, Turkey; 9 Department of Medicine/Division of Rheumatology, Feinberg School of Medicine, Northwestern University Feinberg School of Medicine, Chicago, Illinois, USA; 10 Biobizkaia Health Research Institute, UPV/EHU – University of the Basque Country, Bizkaia, The Basque Country, Spain; 11 Rheumatology Division, Department of Medicine, State University of New York Downstate Health Sciences University, Brooklyn, New York, USA; 12 Division of Rheumatology, Department of Medicine, Queen Elizabeth II Health Sciences Center and Nova Scotia Health; Dalhousie University, Halifax, Nova Scotia, Canada; 13 Division of Rheumatology, Department of Medicine, Dalhousie University, Halifax, Nova Scotia, Canada; 14 Institute of Molecular Medicine, Center for Autoimmune, Musculoskeletal and Hematopoietic Disease, Feinstein Institutes for Medical Research, Northwell Health, Manhasset, New York, USA; 15 Department of Rheumatology, National Reference Center for Autoimmune Diseases (RESO), INSERM UMR-S 1109, Les Hôpitaux Universitaires de Strasbourg, Strasbourg, France; 16 Service de Médecine Interne, Centre de référence des maladies auto-immunes et auto-inflammatoires systémiques rares d'Ile-de-France, de l’Est et de l’Ouest, Hôpital Cochin, AP-HP, Paris, France; 17 Centre for Research in Epidemiology and Statistics, Université Paris Cité; Université Sorbonne Paris Nord; Inserm; INRAE, Paris, France; 18 Copenhagen Lupus and Vasculitis Clinic, Rigshospitalet, Copenhagen University Hospital, Copenhagen, Île-de-France, Denmark; 19 Division of Rheumatology, Department of Medicine, Johns Hopkins University School of Medicine, Baltimore, Maryland, USA; 20 Pediatric Rheumatology Unit, Instituto da Criança e do Adolescente, Hospital das Clínicas da Faculdade de Medicina da Universidade de São Paulo, São Paulo, Brazil; 21 Divisions of Rheumatology & Clinical Epidemiology, Department of Medicine, McGill University Health Centre, Montreal, Quebec, Canada; 22 Department of Rheumatology and Clinical Immunology, Amsterdam University Medical Center, Amsterdam, The Netherlands; 23 Centre for Public Health, Faculty of Medicine, Health and Life Sciences, Queen’s University Belfast, Belfast, Northern Ireland, UK

**Keywords:** Lupus Erythematosus, Systemic, Glucocorticoids, Mortality

## Abstract

**Objective:**

A data-driven and expert/patient consensus-based project to develop a revised Systemic Lupus International Collaborating Clinics (SLICC)/American College of Rheumatology (ACR) Damage Index (SDI) is under way supported by SLICC, ACR, and the Lupus Foundation of America. Our objective is to report the item generation and reduction phase results for a revised SDI.

**Methods:**

Item generation included a literature review by literature review groups and a Delphi exercise of international systemic lupus erythematosus experts and patients. Item reduction involved Delphi rounds in which items with a median appropriateness score of ≤ 4 of 9 were excluded. A 14-member item reduction committee assessed remaining items and removed those that did not reflect the damage construct, were rare, or were not feasible to assess. The clinical domain groups then refined the remaining items and their definitions.

**Results:**

The Delphi panel included 146 individuals from 35 countries. The Delphi exercise nominated 2256 items, and the literature review identified 117 items. After removing redundancies, 226 candidate items remained. Subsequent Delphi rounds, followed by review by the item reduction committee and clinical domain groups, resulted in 39 items across 13 domains. Eleven items from the original SDI, including proteinuria and cranial neuropathy, were removed and several new items were proposed, including growth failure/reduced final height and adrenal insufficiency. Severity-based subitems are proposed for 17 items (43.6%).

**Conclusion:**

This data-driven and expert/patient consensus-based process has proposed 39 candidate items, some with subitems, and definitions for a revised SDI. Weighting of items and subitems is underway to develop a clinical scoring system.

## Introduction

 Systemic lupus erythematosus (SLE) is a chronic autoimmune disease characterised by flares, inflammation in target organs, and increased mortality. Over the past 30 years, there has been a remarkable improvement in the survival rates of patients with SLE due to factors such as earlier diagnosis, improved therapeutic modalities, and better management of complications.^1 2^ Despite the increased longevity in patients with SLE, a range of comorbidities can arise, a number of which are captured as the accrual of organ damage.^3 4^

### Significance & innovations

- The current Systemic Lupus International Collaborating Clinics (SLICC)/American College of Rheumatology (ACR) Damage Index (SDI) is a robust instrument but is limited by missing items, restricted applicability in paediatric patients, a significant floor effect, and outdated item definitions.
- An international initiative is underway to develop a revised SDI using a multiphase process that is supported by SLICC, ACR, and the Lupus Foundation of America.
- Item generation and reduction phases resulted in 39 candidate defined items to be taken forward to the next stages. Subitems based on severity are possible for 17 (43.6%) of the proposed items.
- Our data-driven and expert/patient consensus–based process aims to develop a more detailed and clinically relevant assessment of organ damage in patients with SLE across the lifespan. It will also better reflect current evidence-based medical practice and is likely to improve sensitivity and responsiveness of the index for future lupus trials and research.

Damage in SLE was originally defined as a health state reflecting irreversible changes that occur in patients with SLE and that may be a consequence of ongoing disease activity, treatment (eg, glucocorticoids), comorbidities, or ageing.^5^ Considered a distinct concept from disease activity and health related quality of life, damage serves as a key predictor for morbidity and mortality.^6^ It is therefore crucial to identify patients at risk of damage early enough to implement proactive measures to prevent the onset and progression of damage. In 1996, the Systemic Lupus International Collaborating Clinics (SLICC) group, in collaboration with the American College of Rheumatology (ACR), developed the SLICC/ACR Damage Index (SDI) to quantify irreversible organ damage accrual that occurs in patients with SLE.^5^

The SDI consists of 41 items across 12 organ systems. It captures the accumulation of damage from any cause since SLE diagnosis, regardless of attribution. Generally, items need to be present for 6 months or more to score. Damage may result from disease activity (eg, renal failure or neurocognitive abnormality), treatment (eg, steroid-induced diabetes or avascular necrosis), intercurrent illness (eg, cancer or surgery), or potentially unrelated events (eg, amputation).^5^ Although widely used in SLE cohort studies,^5 6^ the SDI has several limitations.^7^ Its maximum score is 47, but patients rarely exceed 12, and in most cohorts, up to 50% of patients score 0, reflecting a significant floor effect.^8^ The SDI relies on clinical examination and basic investigations. However, the wider use of sophisticated medical investigations means that this approach does not align with modern medical practice. Similarly, many items in the SDI such as chronic kidney disease now have more “staging” in routine practice, which is not reflected well in the often binary scoring (present or absent) in the SDI. From a paediatric viewpoint, the SDI is limited by missing items, for example, growth restriction. Finally, any damage occurring before the formal diagnosis of SLE, even if associated with a prior, but associated, condition such as antiphospholipid syndrome, discoid lupus, or undifferentiated connective tissue disease, would not be counted in the current SDI. Such limitations may contribute to the lower scores observed in cohorts, which limits the usefulness of the SDI as a primary endpoint in SLE trials.

The SLICC group, in collaboration with the ACR and the Lupus Foundation of America (LFA), initiated an international project to revise the SDI using consensus and evidence-based methods over five key phases. In the first phase, we defined the construct of damage in SLE in the modern era using qualitative methods.^9^ By consensus, a contemporary and expanded conceptual framework was agreed on; “Damage is a health state related to organ structure and function. The degree of reduced organ function relates to physiologic impairment. Damage can occur before a diagnosis of SLE but should be attributable to SLE. Damage to an organ is irreversible, but the functional consequences on that organ may improve over time through physiological adaptation or treatment.” In this article, we report the findings of the item generation and reduction phases of the development of the revised SDI.

## Materials and methods

The methodologic approach for development of the revised SDI consists of five stages: updating the construct of damage, item generation, item reduction, item weighting and threshold determination, and assessment of its measurement properties (validity, reliability, and responsiveness). This approach has been successfully used in other rheumatic diseases.^10–13^ Herein, we report the item generation and reduction phases of the revised SDI development. Ethics approval by the University of Manchester was granted on October 13, 2021.

### Item generation

This phase aimed to generate a broad list of candidate items reflecting the new construct of damage in SLE, suitable for inclusion in a new damage index. This process involved a literature review and the first round of a Delphi exercise. Literature review groups conducted targeted, nonsystematic literature searches for each organ domain. Searches were primarily performed in MEDLINE (via PubMed) using domain-specific keywords and relevant terms related to SLE and organ damage (eg, terms derived from the current SDI and organ-specific damage items). These searches aimed to identify relevant reviews, cohort studies, patient surveys, and, where condition-specific evidence was limited, indirect evidence from general population studies. Each domain was subsequently reviewed by paediatric subspecialists for content validity, as it applies to patients with childhood-onset SLE.

Delphi panel members were identified through snowball sampling among SLICC members, in other words, initial investigators were asked to invite others that were known to them. We gave guidance requesting them to consider inviting colleagues from a range of clinical expertise, sex, stages of career, and geographic representation. The LFA, Lupus UK, Lupus Europe, and Lupus Canada were invited to nominate two to three patient/caregiver representatives for the Delphi exercise. The Delphi questionnaires were implemented using Qualtrics software (Qualtrics). In the first round of the Delphi exercise, participants provided demographic and professional details, including profession, country of work, age, sex, race and ethnicity (categories taking into account the global constituency: Arab, Black, Chinese, Korean/Japanese, Latin American, South Asian, Southeast Asian, West Asian, White, or another), primary specialty, and measures of clinical experience (years in practice, average number of patients with SLE seen per month). All participants were asked to nominate items (using their own nomenclature) based on the updated construct definition for inclusion in the revised damage index. Items generated by the literature review groups and by the first Delphi round were reviewed, and repetitive and redundant items were removed.

### Item reduction

Item reduction involved the second and third rounds of the Delphi exercise, evaluation for further refinement of items by an item reduction committee, and clustering based on organ system by the clinical domain groups. In the Delphi rounds, SLE experts and patients rated items on a 9-point Likert scale, considering the new construct of damage in SLE (in which 1 = not at all appropriate and 9 = completely appropriate). In order to ensure equal voice to patient representatives, clear, layperson-friendly definitions of each item were provided to patient representatives. The Delphi process was designed to give equal weight to all contributors, irrespective of whether they were patients or professionals and irrespective of professional discipline or career stage. All ratings and votes were treated equivalently at each round, with no differential weighting applied to patient vs expert responses. In the second Delphi round, participants also were asked to nominate additional items that they felt may have been omitted or were missing. In the third Delphi round, participants were presented with their own ratings and the group median (with 25th–75th percentiles) for each item, allowing them the opportunity to revise their ratings, if appropriate. Items with a median appropriateness rating less than or equal to 4.0 of 9.0 were removed as this score was clearly in the lower range of appropriateness scores. This approach has been successfully used to develop other criteria.^11 13^ Dillman methods (eg, personalised invitations to participate and follow-up reminders) were used to optimise response rates.^14^

Due to insufficient item reduction in the Delphi exercise, an item reduction committee composed of 14 members conducted a thorough assessment of the remaining items. Retained items required a minimum of 10 of 14 (> 70%) agreement to be retained. If an item was rejected by five or more committee members due to any of the following reasons, it was excluded: (1) redundant item, (2) assessment is not widely available or feasible, (3) not reflecting the construct of damage and/or related with lupus disease activity, (4) excessively rare.

Remaining items were then reviewed by expert clinical domain groups for further refinement including whether certain items could be “clustered” into a single item and to identify if any grading of severity could be included for relevant items. These clinical domain groups included an organ system lead who is a rheumatologist, three to four organ-specific specialists, a paediatric rheumatologist, and a patient partner.

### Item groupings and definitions

Our clinical domain groups including rheumatologists and specialty experts reviewed all final items. For each item, they proposed subitem groupings aligned with current clinical practice, such as severity of ventricular dysfunction, staging of chronic kidney disease, and cognitive impairment severity. These groupings were based on contemporary medical literature and routine practice.

Definitions were derived from current evidence and relevant learnt society or professional association guidelines. Where no formal definition existed, the expert group developed one through consensus based on clinical expertise.

A complete list of participants including the leadership team, the literature review groups, the Delphi panel, the item reduction committee, and the clinical domain groups is shown in online supplemental table 3.

## Results

### Delphi panel

We identified 146 individuals from 35 countries, with a mean (range) age of 50.6 (28–79) years. Among the participants, 60.3% were females, and 58.9% identified as White, Latin American (11.0%), Black (7.5%), Korean/Japanese (4.8%), South Asian (4.8%), Arab (3.4%), Chinese (3.4%), Southeast Asian (1.4%), West Asian (0.7%), and another (4.1%). Participants were recruited from a wide range of countries. The largest contributions came from the US (24.0%), Canada (21.2%), and the UK (9.6%), followed by representation from several European, South American, Asian, and African countries. Their clinical experience ranged from 1 to 51 years. This group included 135 medical doctors, 2 allied health professionals, and 9 patients. Of the medical doctors, 120 were rheumatologists, 7 were internists, 5 were nephrologists, 2 were dermatologists, and one was an immunologist (table 1).

**Table 1:** Participant characteristics of the 146 Delphi panel members

Characteristics	N (%)
Participants	
Medical doctor	135 (92.5)
Allied health professional	2 (1.4)
Patient	9 (6.1)
Sex	
Female	88 (60.3)
Male	58 (39.7)
Age, mean (range), y	50.6 (28 – 79)
Race/ethnicity	
White	86 (58.9)
Latin American	16 (11.0)
Black	11 (7.5)
Korean/Japanese	7 (4.8)
South Asian	7 (4.8)
Arab	5 (3.4)
Chinese	5 (3.4)
Southeast Asian	2 (1.4)
West Asian	1 (0.7)
Another	6 (4.1)
Country/region	
US	35 (24.0)
Canada	31 (21.2)
UK	14 (9.6)
European (excluding UK)	27 (18.5)
South America	14 (9.6)
Asia	13 (8.9)
Africa	4 (2.7)
Other North America	3 (2.1)
Caribbean	3 (2.1)
Oceania	2 (1.4)
Main specialty (n = 135)	
Rheumatology	120 (88.9)
Internal medicine	7 (5.2)
Nephrology	5 (3.7)
Dermatology	2 (1.5)
Immunology	1 (0.7)
Clinical experience, median (range), y	20 (1–51)
Main type of practice (n = 135)	
Adults	116 (85.9)
Children	9 (6.7)
Young adults	1 (0.7)
Combination	9 (6.7)
Practice environment (n = 135)	
Teaching hospital	128 (94.8)
Community practice	4 (3.0)
Nonteaching hospital	3 (2.2)

### Item generation

The literature review groups proposed 117 items across 13 clinical domains, including neuropsychiatric, ocular, endocrine/metabolic, growth and development, reproduction/pregnancy, hematologic, gastrointestinal (GI), renal, cardiovascular, peripheral vascular, pulmonary, musculoskeletal, skin/mucocutaneous, and “others.” The response rate of the first round of the Delphi exercise was 97.9% and resulted in 2,256 “raw” proposed items. After the first Delphi round, we reviewed all nominated items, clustered overlapping items, and harmonised synonymous terms. For example, stroke was proposed by many participants, and some used the term “cerebrovascular accident.” All of these were reduced to a single item. This process led to the removal of 2,040 “raw” items. We then compared the remaining items with those identified through the literature review. All items in the original SDI were nominated in both processes. We found that the first Delphi round generated 103 unique items (46.8%), whereas the literature review generated four unique items (1.8%), and 113 items (51.4%) appeared in both exercises (online supplemental table 2). In total, 220 discrete proposed items were retained.

In the second Delphi round, participants suggested 53 potential additional “raw” items; however, only six were retained, as 47 others were deemed to be duplicates or synonyms of existing proposed items. These new items included intracranial hypertension, disordered puberty sequences, limb length discrepancies, low anti-Müllerian hormone levels, lipodystrophy, and alopecia without scarring. In total, 226 unique items were generated at this stage.

### Item reduction

The response rates for the second and third rounds of the Delphi exercise were 95% and 91.7%, respectively. There was stability in median scores and the 25th to 75th percentiles for each item between the second and third rounds. All original SDI items (with two exceptions) were retained with group median appropriateness scores of 7 to 9; “upper GI tract surgery” and “osteomyelitis” were retained with median appropriateness scores of 5. In the third Delphi round, 36 items had a median score of 4.0 or less, thus reducing the items from 226 to 190 (table 2).

**Table 2:** Summary median scores after the third round of Delphi exercise (in which 1=not at all appropriate and 9=completely appropriate)*

Candidate items	Median
Neuropsychiatric	
Ischaemic or haemorrhagic stroke	9
Transverse myelitis	9
Cognitive impairment	8
Cranial neuropathy	8
Neurological sequelae (any) from neuropsychiatric SLE	8
Peripheral neuropathy	8
Seizure disorder	8
Dementia	7
Demyelinating disorder including multiple sclerosis	7
Movement disorders (ataxia, tremors, chorea, hemiballismus)	7
Psychosis	7
Executive function disorder	6
Radiographically proven brain atrophy	6
Autonomic disorder	5
Cognitive dyslexia	5
Encephalitis	5
Image changes captured by functional MRI or SPECT	5
Incapacitating headaches	5
Intracranial hypertension	5
Hearing impairment	4.5
Loss of sensation	4.5
Delirium	4
Mood disorder	4
Attempted suicide	3
Insomnia	3
Poor scholastic achievement	3
Tinnitus	3
Ocular	
Chloroquine and hydroxychloroquine maculopathy	9
Cataract	8
Optic atrophy	8
Permanent uniocular or binocular partial or complete loss of vision	8
Retinal necrosis and/or retinal detachment (choroidopathy, lupus retinopathy, serous retinopathy, retinal thrombosis)	8
Corneal scarring/corneal ulceration	7
Proliferative retinopathy	7
Scleral thinning (as a result of anterior scleritis)	7
Chronic angle closure, open angle glaucoma, and steroid-induced glaucoma	6
Cicatricial ectropion (from DLE)	6
Madarosis (from DLE)	6
Optic neuropathy (due to ischemia or inflammation)	6
Persistent uveitis or scleritis	6
Retinal depigmentation and functional loss	6
Cicatricial conjunctivitis or symblepharon	5
Keratoconjunctivitis sicca	5
Acquired enophthalmos	4
Endocrine/metabolic	
Premature ovarian failure/primary ovarian insufficiency	9
Steroid-induced diabetes	8
Adrenal insufficiency	7
Diabetes mellitus	7
Arterial hypertension	6
Cushing syndrome	6
Metabolic syndrome	6
Reduced ovarian reserve	6
Testicular dysfunction with reduced sperm count and quality/gonadal dysfunction	6
Cachexia/wasting	5
Chronic fatigue syndrome	5
Erectile dysfunction	5
Hyperlipidemia	5
Hypopituitarism	5
Persistent weight gain	4
Anti-insulin receptor antibodies with recurrent hypoglycaemia	3
Eating disorder	3
Hyperthyroidism	3
Hypothyroidism	3
Facial and/or extremity swelling	2
Growth and development	
Growth failure	8
Nontraumatic long bone fractures for children	8
Delayed puberty	7
Reduced final height	7
Disordered puberty sequences, eg, reaching menarche without/with minimal/less than expected growth spurt and breast or pubic hair development	6
Limb length discrepancies	5
Reproduction/pregnancy	
Congenital heart block	7
Hypertensive disorders of pregnancy (gestational hypertension, preeclampsia, and/or eclampsia)	7
Infertility	6
Intra-uterine growth restriction, small for gestational age	6
Miscarriage/fetal loss	6
Obstetric antiphospholipid syndrome	6
Preterm birth	6
Stillbirth	6
Cervical dysplasia	5
Gestational diabetes	5
Low anti-Müllerian hormone levels	5
Neonatal lupus in offspring	4
Placental abruption	4
Hematologic	
Splenectomy (for ITP)	8
Aplastic anaemia	7
Bone marrow failure (aplasia, myelodysplasia, or myelofibrosis)	7
non-Hodgkin's lymphoma	7
Spleen infarction	7
Treatment-related myelodysplastic syndrome	7
Antiphospholipid syndrome	6
Functional asplenia	6
Prolonged cytopenia	6
Splenic atrophy	6
Chronic anaemia	5
Thrombocytopenia, leucopenia	5
Keloid scar linked to lymph node sampling	3
Gastrointestinal	
Bowel stricture	7
Budd-Chiari syndrome	7
Chronic ascites	7
Chronic liver disease and cirrhosis including nodular regenerative hyperplasia	7
Chronic peritonitis	7
End-stage liver disease	7
Malabsorption and protein losing enteropathy	7
Mesenteric insufficiency	7
Pancreatic insufficiency or need to use pancreatic enzyme replacement	7
Surgical resection of bowel below the duodenum, spleen, liver, or gallbladder	7
Vasculitis of the mesentery, oesophagus, stomach, peritoneum, gallbladder, pancreas, or liver resulting in organ necrosis	7
Gastroparesis	6
Motility disorders including oesophageal dysmotility and pseudo–bowel obstruction	6
Dysphagia	5
Myositis with dysphagia	5
Upper gastrointestinal tract surgery	5
Cholecystitis	3
Gastrointestinal bleed	3
Mesenteric adenitis	3
Bariatric surgery	2
Renal	
Chronic kidney disease	9
Decreased eGFR	9
End-stage renal disease	9
Renal biopsy findings (chronicity index > 4 OR interstitial fibrosis > 25% OR tubular atrophy > 25%)	8
Biopsy proven renal microangiopathy due to antiphospholipid syndrome even without GFR less than 50%	7
Chronic subnephrotic proteinuria	7
Proteinuria	7
Renal infarction	7
Renal tubular defect	7
Bladder dysfunction	5
Haemorrhagic cystitis	5
Nephritic syndrome	4
Hematuria	3
Renal calculi	3
Sterile pyuria	2
Cardiovascular	
Cardiomyopathy (ventricular dysfunction)	9
Myocardial infarction ever	9
Accelerated and early atherosclerosis	8
Angina or coronary artery bypass	8
Pericarditis, pericardial thickening, or pericardiectomy	8
Pulmonary hypertension	8
Valve vegetations	8
Carotid endarterectomy/stenting	7
Complete auriculoventricular block	7
Conduction system disease	7
Myocardial fibrosis	7
Right ventricular dysfunction	7
Valve stenosis	7
Valve thickening	7
Arrhythmias	6
Arterial hypertension	6
Baseline oxygen requirement	6
Diastolic dysfunction	6
Valve regurgitation	6
Aortitis	5
Cardiogenic shock not due to sepsis	5
QT prolongation	5
Peripheral vascular	
Significant tissue loss ever (eg, loss of digit or limb)	9
Claudication	7
Intracardiac thrombus	7
Minor tissue loss (pulp space)	7
Renal artery stenosis	7
Severe Raynaud phenomenon	7
Thrombosis any (arterial or venous)	7
Venous thrombosis or venous stasis	7
Pulmonary	
Pulmonary fibrosis	9
Pulmonary hypertension	9
Pleural fibrosis, pleurodesis, or pleurectomy for refractory effusions	8
Pulmonary infarction or resection not for malignancy	8
Chronic diffuse alveolar haemorrhage	7
Lobectomy	7
Persistently decreased exercise tolerance/DLco due to ILD or pulmonary emboli	7
Respiratory insufficiency	7
Restrictive lung disease (based on pulmonary function test abnormalities)	7
Diminished lung function (decreased DLco, FEV_1_, FVC)	6
Bronchiectasis	5
Obstructive airway disease	4
Obstructive sleep apnea	4
Musculoskeletal	
Avascular necrosis/Legg-Calve-Perthes disease/Perthes disease	9
Osteoporosis with fracture	9
Erosive arthritis	8
Fragility fractures	8
Jaccoud arthropathy	8
Joint replacement	8
Atrophy or weakness	7
Muscle atrophy	7
Osteoporosis without fracture	7
Tendon rupture	7
Osteomyelitis	6
Sarcopenia	6
Frailty	5
Osteomalacia	4
Fibromyalgia	3
Osteosclerosis	3
Septic arthritis	3
Chronic gout with erosion and tophi	2
Inflammatory joint pain	2
Skin/mucocutaneous	
Scarring chronic alopecia	9
Scarring (cutaneous)	8
Skin ulceration (excluding thrombosis) for >6 mo	8
Chronic discoid or subacute lesions	7
Cicatricial skin atrophy	7
Hyperpigmentation or hypopigmentation	7
Post venous thrombosis complications	7
Calcinosis	6
Extensive striae	6
Panniculitis	6
Alopecia without scarring	5
Hirsutism	5
Lipodystrophy	5
Nasal septum perforation	5
Periodontal disease with tooth loss	5
Persistent malar rash	5
Severe livedo reticularis	5
Severe photosensitivity limiting activities of daily living	5
Sjögren syndrome	5
Telangiectasia	5
Xerostomia	5
Easy bruisability	3
Onychodystrophy	3
Others	
Malignancy	8
Organ transplantation (nonkidney)	8
Treatment-related damage	8
Persistent hypogammaglobulinemia	7
Severe or recurrent infections	7
Carcinoma in situ (any site)	6
Work disability	6
Social isolation	4
Poverty	3

DLco, diffusing capacity for carbon monoxide; DLE, discoid lupus erythematosus; DLE, discoid lupus erythematosus; eGFR, estimated glomerular filtration rate; FEV1, forced expiratory volume in 1 second; FVC, forced vital capacity; ILD, interstitial lung disease; ITP, immune thrombocytopenia; MRI, magnetic resonance imaging; SLE, systemic lupus erythematosus; SPECT, single-photon emission computerized tomography.

The item reduction committee then assessed the remaining 190 items, and we excluded items that were rejected by five or more members of the committee (ie, a 70% threshold to retain) due to at least one of the following: redundancy, ascertainment not widely available or feasible, items not reflecting the construct of damage, and excessively rare items (online supplemental table 3). Consequently, 126 items were removed from the candidate item list.

**Figure 1:**
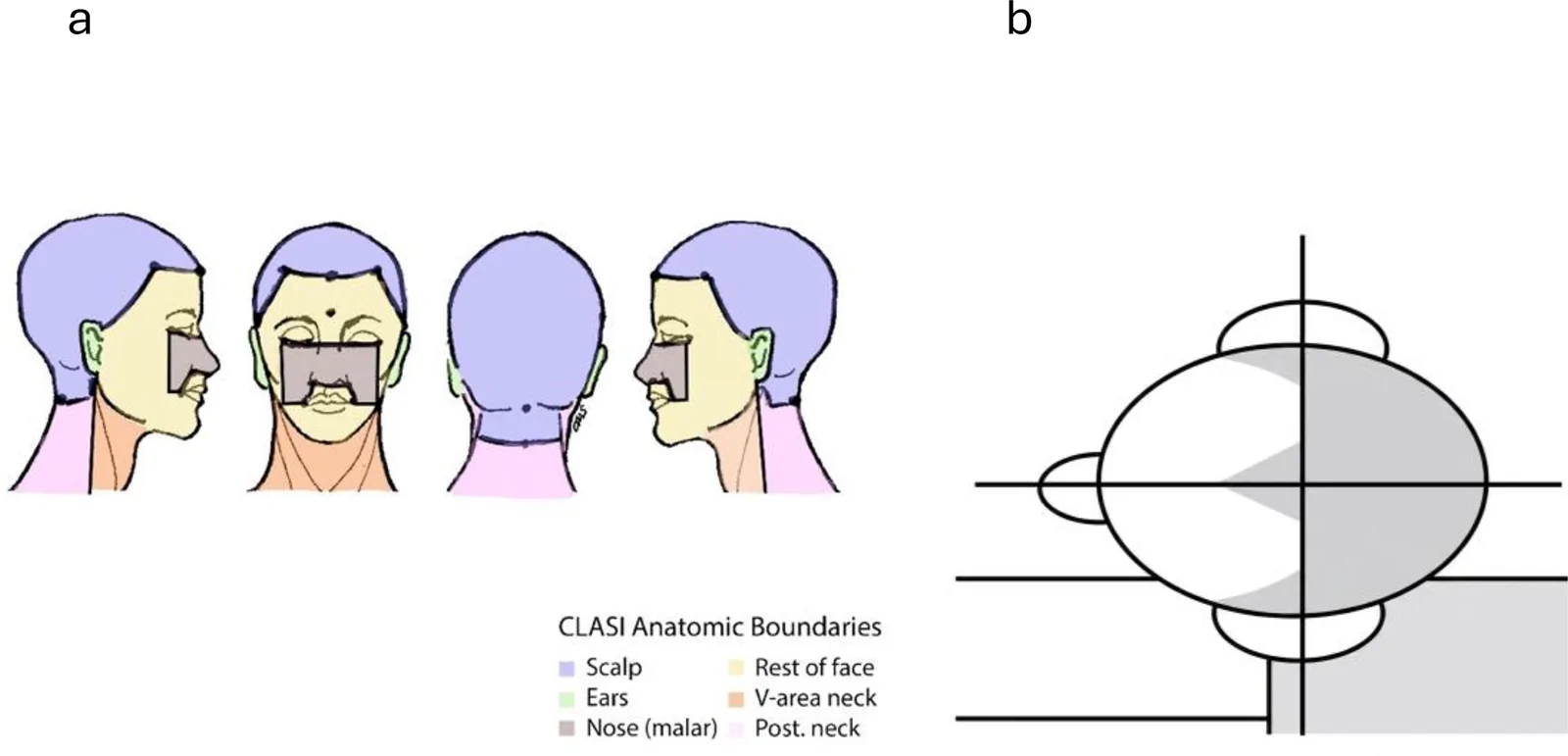
Anatomic boundaries of (a) the scalp and (b) the four quadrants of the scalp using the CLASI boundaries.^74^ CLASI, Cutaneous Lupus Erythematosus Disease Area and Severity Index. Figure (b) is AI generated using Google Gemini.

**Figure 2:**
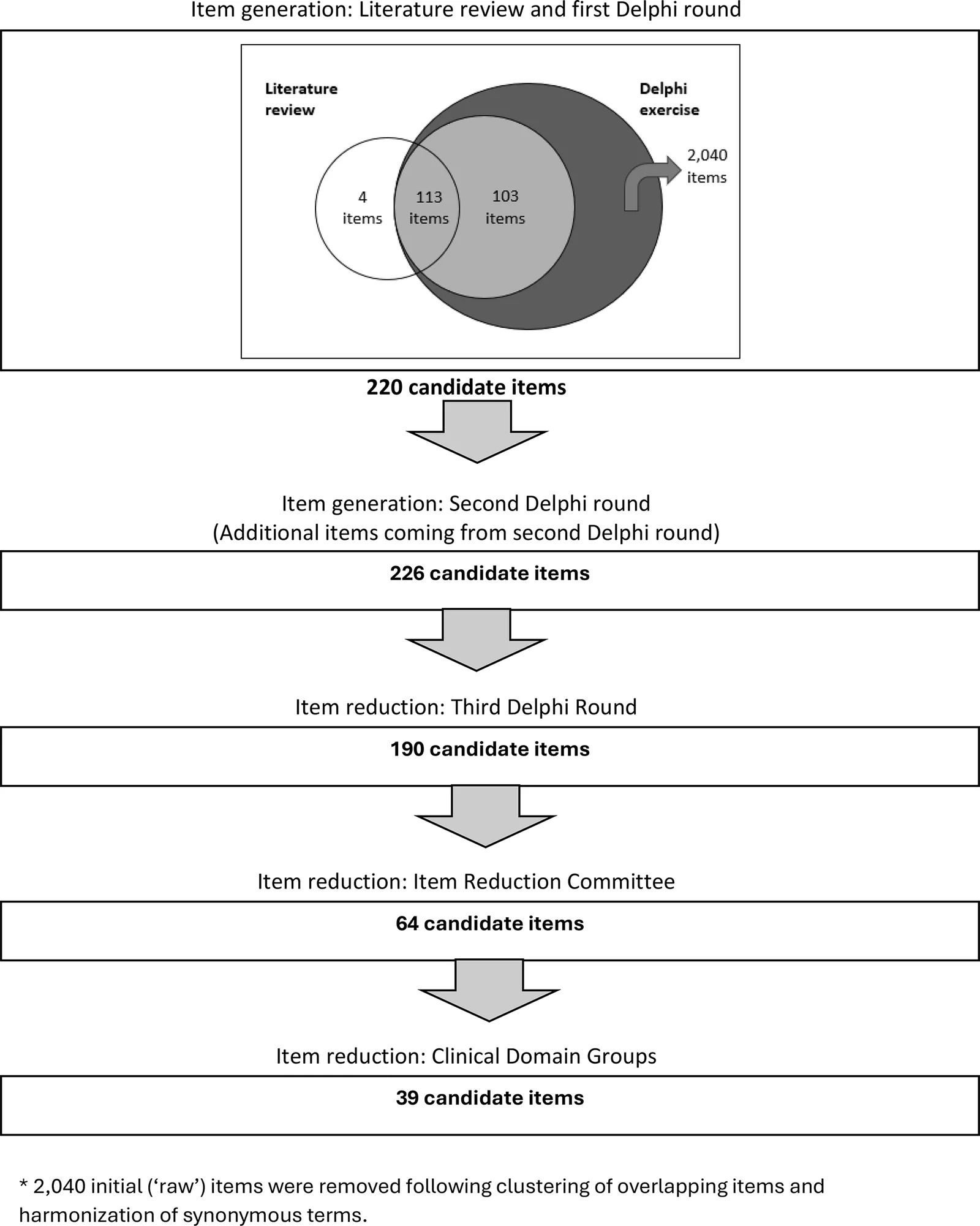
Number of candidate items identified during the item generation and item reduction phases of the revised SDI development. SDI, Systemic Lupus International Collaborating Clinics/American College of Rheumatology Damage Index.

The remaining 64 items were re-evaluated by the clinical domain groups. Items such as cranial neuropathy, psychosis, and movement disorders were deemed incongruent with the current construct of damage and were consequently removed, as they were more reflective of SLE disease activity. Similarly, very rare items such as bowel stricture, renal infarction, pleural fibrosis, and skin ulceration were also excluded as individual stand-alone items. The clinical domain groups also identified overlapping items and recommended clustering them into single items for clarity and efficiency. For instance, dementia and cognitive impairment were merged into a single item due to them being distinguished only by degree of severity. The items in the hematologic domain overlapped with other clinical domains, and so the domain was removed. (Online supplemental table 4) presents the excluded items and their reasons for exclusion by the clinical domain groups. These item reduction phases resulted in 39 proposed candidate items across 13 clinical domains with glossary definitions developed by the clinical domain groups (table 3 and figure 1) and a draft index developed (table 4). Figure 2 summarises the number of candidate items that were generated and reduced.

**Table 3:** Proposed revised lupus damage index glossary (score only the worst outcome for each item)*

Domain, item, and subitem	Definition and duration
Neuropsychiatric	
Ischaemic or haemorrhagic stroke	
Asymptomatic (imaging only)	Imaging evidence (CT or MRI) of infarct or haemorrhage without an associated clinical syndrome. ** Duration: once item definition is met**.
Single symptomatic stroke	Acute focal neurologic deficit persisting more than 24 hours with a corresponding CT or MRI abnormality (infarct or haemorrhage) consistent with the clinical syndrome. **Duration: once item definition is met**.
More than one stroke	Evidence of multiple strokes at different anatomic sites. This item includes ischaemic or haemorrhagic spinal cord events. Nonspecific white matter changes on MRI are not considered organ damage. This definition is adapted from the 1999 ACR nomenclature.^33^**Duration: once item definition is met**.
Demyelinating disease (including transverse myelitis)	Monophasic or relapsing CNS demyelinating syndrome, based on a combination of clinical findings typical for CNS demyelination and supportive abnormalities on neuroimaging (typically MRI).^34^ Includes transverse myelitis,^34^ multiple sclerosis,^35^ neuromyelitis optica spectrum disorders,^36^ SLE-related demyelination, and other causes of demyelinating disease. Isolated optic neuritis is excluded here as any visual loss will be captured elsewhere (see Ocular domain). **Duration: 6 mo**.
Cognitive impairment	Evidence of cognitive decline from a previous level of performance in one or more cognitive domain (complex attention, executive function, learning and memory, language, perceptual motor, or social cognition) based on the following: concern of the individual, a knowledgeable informant, or the clinician that there has been a significant decline in cognitive function AND a substantial impairment in cognitive performance, preferably documented by standardised neuropsychological testing or, in its absence, another quantified clinical assessment.^37^**Duration: 6 mo**.
Mild neurocognitive impairment	Cognitive deficits are insufficient to interfere with independence in everyday activities (eg, instrumental activities of daily living) but greater effort, compensatory strategies, or accommodation may be required. **Duration: 6 mo**.
Major neurocognitive impairment	Cognitive deficits are sufficient to interfere with independence in everyday activities (eg, instrumental activities of daily living). Cognitive deficits must not occur exclusively in the context of a delirium. **Duration: 6 mo**.
Peripheral neuropathy	Polyneuropathy is a disorder of sensory and motor peripheral nerves characterised by symmetry of symptoms and physical findings in a distal distribution.^33^ Mononeuropathy (single/multiplex) is the disturbed function of one or more peripheral nerves resulting in weakness or sensory dysfunction due to either conduction block in motor fibres or axonal loss.^33^ Requires clinical demonstration of sensory and/or motor deficits on neurologic examination with corresponding abnormalities on electromyography and/or nerve conduction studies.^33^ Small fibre neuropathy is not included as objective testing methods for confirmation of this diagnosis are not readily available in most centres. **Duration: 6 mo**.
Seizure disorder	Recurrent unprovoked seizures (defined as two or more, at least 24 hours apart) with ongoing high probability of recurrence OR a single unprovoked seizure with a high probability of recurrence based on abnormalities identified on brain MRI and/or EEG. Isolated seizure occurring in the context of active SLE is not considered organ damage. Use of anticonvulsant therapy should not be included in the item definition, as this is highly dependent on physician practice patterns and other contextual factors. **Duration: 6 mo**.
Ocular	
Cataract or antimalarial maculopathy identified without objective visual loss	Cataract and/or antimalarial maculopathy identified *without objective visual loss*. **Duration: 6 mo**.
ANY ocular disease resulting in the following:	
Partial visual loss affecting the peripheral visual field alone (uniocular or binocular)	Partial visual loss affecting the peripheral visual field alone (uniocular or binocular). **Duration: 6 mo**.
Partial visual loss affecting the central visual field alone (uniocular or binocular)	Partial visual loss affecting the central visual field alone (uniocular or binocular). **Duration: 6 mo**.
Uniocular complete visual loss affecting both central and peripheral visual fields	Uniocular complete visual loss affecting both central and peripheral visual fields. **Duration: 6 mo**.
Binocular complete visual loss affecting both central and peripheral visual fields	Binocular complete visual loss affecting both central and peripheral visual fields. **Duration: 6 mo**. Score the worst outcome/stage for the patient. See references.^38–40^
Endocrine	
Premature Ovarian Insufficiency (POI)	POI is the onset of hypergonadotropic hypogonadism before the age of 40 y due to any cause. Criteria for diagnosis: irregular menses for greater than three mo without another endocrine disorder (thyroid hormonal dysfunction, hyperprolactinemia) AND elevated FSH > 30 mIU/mL on two measurements assessed at least one mo apart. Some women with POI present with infertility.^31 32^**Duration: more than three mo**.
Diabetes mellitus, including steroid-induced diabetes	Hyperglycaemia that fulfils any of the following criteria: (a) fasting plasma glucose ≥ 126 mg/dL (7 mmol/L), (b) 2 hour plasma glucose ≥ 200 mg/dL (11.1 mmol/L) with an oral glucose tolerance test, (c) presence of osmotic symptoms (eg, polyuria, polydipsia) of hyperglycaemia with a random plasma glucose ≥ 200 mg/dL (11.1 mmol/L), or (d) HbA_1c_ ≥ 6.5.^41^ HbA_1c_ will not be elevated in acute hyperglycaemia. **Duration: 6 mo**.
Adrenal insufficiency	Inadequate cortisol secretion by the adrenal glands. Low early morning (6–9 am) basal serum cortisol concentration (≤ 3 mcg/dL(80 nmol/L)) is consistent with adrenal insufficiency. Early morning basal serum cortisol concentration (≥13 to 14 mcg/dL(≥360 to 390 nmol/L)) rules out adrenal insufficiency. When the morning basal serum cortisol concentration is >3 mcg/dL (>80 nmol/L) but< 13 to 14 mcg/dL (< 360 to 390 nmol/L), a standard ACTH stimulation test should be performed. Peak serum cortisol after ACTH stimulation ≥ 18 mcg/dL (500 nmol/L) rules out adrenal insufficiency.^42–45^**Duration: 6 mo**.
Cushing's syndrome	Cushing's syndrome is diagnosed clinically with typical clinical and phenotypic features in a patient taking chronic glucocorticoid therapy. If endogenous Cushing syndrome is present, then confirmation by specialist investigations is required.^46–48^**Duration: 6 mo**.
Testicular dysfunction with reduced sperm counts and quality	Low serum testosterone concentration and/or sperm count associated with elevated serum luteinising hormone and/or FSH concentrations. Fasting serum total testosterone should be assessed between 8 and 10 am on at least two occasions).^49^**Duration: 6 mo**.
Growth and development	
Growth failure or reduced final height	Final height lower than the midparental range or ≤ 2 SD under the mean race/sex-adjusted height as per national growth charts or the WHO growth chart when measured first at age 18 y and again at or after age 20 y. **Duration: once item definition is met**.
Gastrointestinal	
Surgical resection of bowel below theduodenum, spleen, liver, or gallbladder:	Surgical resection (documented in the medical record) of bowel below the duodenum, spleen, liver, or gallbladder. **Duration: once item definition is met**.
1 site More than 1 site	
Motility disorders including oesophageal dysmotility and pseudo–bowel obstruction	Oesophageal dysmotility or pseudo-obstruction are defined by a combination of typical clinical symptoms and confirmatory testing including relevant imaging and motility/manometry studies.^50^ If due to malignancy, do not score the item here; instead use the Malignancy domain. **Duration: 6 mo**.
Renal	
Renal function impairment:	Renal function impairment is defined according to the KDIGO categories^15^ of estimated GFR: eGFR > 90 (G1), eGFR 60–89 (G2), eGFR 45–59 (G3a), eGFR 30–44 (G3b), eGFR 15–29 (G4), or eGFR < 15 (G5). **Duration: renal function impairment must persist for 12 mo or more. Dialysis must be present for six mo or more. Transplantation once item definition is met**.
eGFR 30–59 (G3a–G3b) eGFR 15–29 (G4), eGFR < 15 (G5) OR hemo/peritoneal dialysis OR renal transplantation	
Chronicity on renal biopsy:	Renal biopsy findings are scored according to modified NIH activity and chronicity indices.^16^ **Duration: once item definition is met**.
Chronicity index 2–4 Chronicity index > 4	
Cardiovascular	
Cardiomyopathy (ventricular dysfunction):	Cardiomyopathy (ventricular dysfunction) is defined using echocardiographic documentation of LVEF^51^:
Heart failure with preserved EF (≥50%)	LVEF≥50% with evidence of spontaneous or provokable increased LV filing pressures (eg, elevated natriuretic peptides, noninvasive and invasive haemodynamic measurement). **Duration: 6 mo**.
Heart failure with mildly reduced EF (41%–49%)	LVEF 41%–49%, measured by echocardiogram, with evidence of spontaneous or provokable increased LV filing pressures (eg, elevated natriuretic peptides, noninvasive and invasive haemodynamic measurement). **Duration: 6 mo**.
Heart failure with reduced EF (≤40%)	LVEF ≤ 40%, measured by echocardiogram. **Duration: 6 mo**.
Heart failure with improved EF	Previous LVEF ≤ 40% with follow-up > 40% and improvement persisting for six mo or more. **Duration: 6 mo**.
Ischaemic heart disease	
Asymptomatic ischemia	Asymptomatic ischemia as identified on any of the following: EKG and/or stress testing and/or cardiac MRI. ** Duration: 6 mo**.
Symptomatic angina	Symptomatic angina as confirmed by EKG and/or stress test and/or cardiac catheterization and/or cardiac MRI. **Duration: 6 mo**.
MI and/or stenting in one coronary artery or one region of a single artery	MI documented by clinical presentation and confirmatory EKG evidence and positive troponins and/or stenting in one coronary artery or one region of a single artery. **Duration: once item definition is met**.
Multiple MIs and/or coronary artery by-pass grafting and/or stenting in two separate regions of 1 artery or two or more arteries	Multiple MIs and/or coronary artery by-pass grafting/stenting in two separate regions of 1 artery or two or more arteries. **Duration: once item definition is met**.
Vegetations or thickening on any valve	Based on the American Society of Echocardiography guidelines,^52^ valve vegetation is defined as shaggy, lobulated, or rounded masses typically located on the atrial side of atrioventricular valves (mitral valve and tricuspid valve) or ventricular side of the aortic valve but can be located on any side of any valve. These can occur as the following:
Vegetations/thickening without valvular dysfunction	Vegetations/thickening without valvular dysfunction. **Duration: 6 mo**.
Vegetations/thickening with mild-moderate valvular dysfunction	Vegetations/thickening with mild-moderate valvular dysfunction. **Duration: 6 mo**.
Vegetations/thickening with severe valvular dysfunction OR valve replacement	Vegetations/thickening with severe valvular dysfunction OR valve replacement. **Duration: 6 mo**.
Pulmonary hypertension:	
Pulmonary hypertension with right heart catheterization showing mean pulmonary artery pressure > 20 mm Hg or echocardiogram showing estimated pulmonary artery systolic pressure > 40 mm Hg	Right heart catheterization showing mean pulmonary artery pressure > 20 mm Hg or echocardiogram showing estimated pulmonary artery systolic pressure > 40 mm Hg. **Duration: 6 mo**.
Pulmonary hypertension with cor pulmonale	Right heart catheterization showing mean pulmonary artery pressure > 20 mm Hg or echocardiogram showing estimated pulmonary artery systolic pressure > 40 mm Hg AND evidence of alteration of the right heart structure and function due to pulmonary hypertension. See reference.^53^ **Duration: 6 mo**.
Pericarditis, pericardial thickening, or pericardiectomy	Chronic and persisting signs and symptoms of pericarditis and/or pericardial thickening demonstrated on imaging. Surgical removal of part or all of the pericardium surrounding the heart. **Duration: 6 mo**.
Carotid endarterectomy/stenting	Insertion of an expandable wire mesh tube or coil into the artery to maintain blood flow. **Duration: 6 mo**.
Vascular	
Arterial thrombosis including organ infarcts other than MI and stroke	Arterial thrombosis with organ infarction of any organ other than the heart or brain. ** Duration: once item definition is met**.
Peripheral ischemia/gangrene withtissue loss:	Ischemia and/or gangrene with tissue loss due to involvement of a specific vascular structure (artery, vein). Imaging studies such as doppler, or ankle-brachial index test, to identify the vascular structure is recommended but not required for inclusion.^54^**Duration: 6 mo**.
1 site More than one site	
Peripheral arterial disease	Objective evidence of peripheral arterial vascular insufficiency demonstrated by ankle-brachial doppler testing or angiography with or without classic symptoms of intermittent claudication without ischaemic damage or gangrene. **Duration: 6 mo**.
Post–venous thrombosis complications	Extremity swelling, skin discolouration, nonhealing ulceration, and/or amputation. **Duration: 6 mo**.
Pulmonary	
Pulmonary Fibrosis (PF):	
Mild or moderate PF	Evidence for PF due to any cause^55–58^ by HRCT (less than 20% of lung areas) at any time AND impaired PFT with FVC 50%–80% that persists for at least six mo. **Duration: 6 mo**.
Advanced PF	Clinical evidence (fine crackles) and/or extensive fibrosis, due to any cause, identified by chest radiograph^29^ OR evidence for advanced PF by HRCT (> 20% of lung areas) OR impaired PFTs with FVC < 50%. **Duration: 6 mo**.
Pulmonary infarction or resection not for malignancy	Peripheral wedge-shaped consolidation with a convex medial border on x-ray^59^ OR CT angiogram (peripheral wedge-shaped broad pleural parenchymal density). **Duration: once item definition is met**.
Chronic dyspnoea	Chronic dyspnoea not related to daily activity with severely impaired PFTs without advanced PF due to any cause including pathology related to the lungs and pleura. Examples include shrinking lung (diaphragmatic elevation by x-ray and/or >20% decrease of FVC in supine position) and pleural fibrosis (x-ray or CT).^56 60 61^**Duration: 6 mo**.
Musculoskeletal	
Avascular necrosis:	Regional death of cellular elements of bone, typically in a subchondral location, due to a disruption in the blood supply leading to ischemia, infarction.^62–64^**Duration: once item definition is met**.
1 site More than 1 site	
Osteoporosis with fracture or nontraumatic long bone fractures for children	Low-energy bone fracture or spinal fracture or nontraumatic long bone fractures for children with radiologic evidence of bone loss on bone densitometry.^65^**Duration: once item definition is met**.
Erosive arthropathy	Loss of mineralized tissue at marginal or juxta-articular sites, commonly associated with a break in the cortical lining visualised on plain radiographs, CT, or MRI or demonstrated on two planes on ultrasonography.^66 67^**Duration: 6 mo**.
Jaccoud arthropathy	Reducible joint deformities such as swan neck, thumb subluxation, ulnar deviation, “boutonniere,” and Z-thumb, with or without evidence of erosions on plain radiograph.^68 69^**Duration: 6 mo**.
Joint replacement	Elective replacement of any joint(s) due to joint or bone diseases, regardless of the SLE disease activity at the time of joint replacement.^70 71^**Duration: once item definition is met**.
Tendon rupture	Atraumatic rupture of one or more tendons regardless of SLE disease activity at the time of diagnosis of tendon rupture.^72 73^**Duration: once item definition is met**.
Skin/mucocutaneous	
Cutaneous scarring or atrophy (including lipoatrophy):	Cutaneous scarring or atrophy (including lipoatrophy): in defining this item, the anatomic location and extent should be noted. The scalp is *excluded* from this item; see figure 1a. **Duration: 6 mo**.
Affecting the trunk and/or extremities Affecting the head and/or neck(excluding the scalp, Figure 1a)	
Dyspigmentation:	May be hyperpigmentation or hypopigmentation and duration of changes should be considered when scoring this item.
More than six mo but less than 12 mo duration More than 12 mo duration	
Scarring chronic alopecia	The scalp anatomic boundaries are the natural hairline (follicles present) OR, if bald, the frontal border begins 8 cm from the midpoint of the glabella and extends 8 cm left and right from that midpoint. For the back of the head, the theoretical hairline would end at 8 cm below the external occipital protuberance (figure 1a).^74^ Divide the scalp into four quadrants as shown in figure 1b.^74^ **Duration: 6 mo**.
1 quadrant 2 quadrants 3–4 quadrants	
Malignancy	
Malignancy:	Any malignancy diagnosed by a combination of clinical, imaging, and histologic features that confirm cancer. ** Duration: once item definition is met**.
Non-melanoma skin cancer Any other primary malignancy Two or more malignancies	
Organ transplantation	
Organ transplantation (nonkidney)	Transplantation of any organ apart from the kidney (see Renal domain). **Duration: once item definition is met**.

* Items and subitems are presented in their relevant clinical domains. Items with subitems are defined to reflect current guidelines and/or clinical expert opinion. Items and subitems need to be present for a minimum of 6 mo unless otherwise stated in bold. Acute events can be defined once the item occurs. Items occurring after the diagnosis of SLE DO NOT need to be directly attributed to SLE. Items occurring before the diagnosis of SLE should be scored if attributable to an SLE-related process† by the assessor. Only score the highest scoring subitem for each item

† Examples of an “SLE-related process” before the diagnosis of SLE could include manifestations of antiphospholipid syndrome, interstitial lung disease due to a disease process that evolves into SLE, or deforming inflammatory arthritis that evolves into SLE.

ACR, American College of Rheumatology; ACTH, adrenocorticotropic hormone; CNS, central nervous system; CT, computed tomography; EEG, electroencephalography; EF, ejection fraction; eGFR, estimated glomerular filtration rate; EKG, electrocardiogram; FSH, follicle-stimulating hormone; FVC, forced vital capacity; GFR, glomerular filtration rate; HbA_1c_, glycosylated hemoglobin; HRCT, high resolution computerized tomography; KDIGO, Kidney Disease Improving Global Outcomes; LV, left ventricular; LVEF, left ventricular ejection fraction; MI, myocardial infarction; MRI, magnetic resonance imaging; PF, pulmonary fibrosis; PFT, pulmonary function test; POI, premature ovarian insufficiency; SLE, systemic lupus erythematosus.

**Table 4:** Patient assessment sheet for proposed revised lupus damage index*

Domain	Item and subitem	
Neuropsychiatric	Ischaemic or haemorrhagic stroke:	
	Asymptomatic (imaging only) Single symptomatic stroke More than one stroke	□ Yes □ No □ Yes □ No □ Yes □ No
	Demyelinating disease (including transverse myelitis)	□ Yes □ No
	Cognitive impairment:	
	Mild neurocognitive impairment Major neurocognitive impairment	□ Yes □ No □ Yes □ No
	Peripheral neuropathy	□ Yes □ No
	Seizure disorder	□ Yes □ No
Ocular	Ocular damage:	
	Cataract or antimalarial maculopathy identified without objective visual loss	□ Yes □ No
	ANY ocular disease resulting in the following:	
	Partial visual loss affecting the peripheral visual field alone (uniocular or binocular) Partial visual loss affecting the central visual field alone (uniocular or binocular) Uniocular complete visual loss affecting both central and peripheral visual fields binocular) Binocular complete visual loss affecting both central and peripheral visual fields	□ Yes □ No □ Yes □ No □ Yes □ No □ Yes □ No
Endocrine	Premature ovarian insufficiency	□ Yes □ No
	Diabetes mellitus, including steroid-induced diabetes	□ Yes □ No
	Adrenal insufficiency	□ Yes □ No
	Cushing syndrome	□ Yes □ No
	Testicular dysfunction with reduced sperm counts and quality	□ Yes □ No
Growth and development	Growth failure or reduced final height	□ Yes □ No
Gastrointestinal	Surgical resection of bowel below the duodenum, spleen, liver, or gallbladder:	
	one site More than one site	□ Yes □ No □ Yes □ No
	Motility disorders including oesophageal dysmotility and pseudo–bowel obstruction	□ Yes □ No
Renal	Renal function impairment:	
	eGFR 30 – 59 (G3a – G3b) eGFR 15 – 29 (G4) eGFR < 15 (G5) OR hemo/peritoneal dialysis OR renal transplantation	□ Yes □ No □ Yes □ No □ Yes □ No
	Chronicity on renal biopsy:	
	Chronicity index (2– 4) Chronicity index (> 4)	□ Yes □ No □ Yes □ No
Cardiovascular	Cardiomyopathy (ventricular dysfunction):	
	Heart failure with preserved EF (≥ 50%) Heart failure with mildly reduced EF (41% – 49%) Heart failure with reduced EF (≤ 40%) Heart failure with improved EF (previously ≤ 40% with follow-up > 40%)	□ Yes □ No □ Yes □ No □ Yes □ No □ Yes □ No
	Ischaemic heart disease:	
	Asymptomatic ischemia Symptomatic angina MI and/or stenting in one coronary artery or one region of a single artery Multiple MIs and/or coronary artery by-pass grafting and/or stenting in two separate regions of 1 artery or two or more arteries	□ Yes □ No □ Yes □ No □ Yes □ No □ Yes □ No
	Vegetations or thickening on any valve:	
	Vegetations/thickening without valvular dysfunction Vegetations/thickening with mild-moderate valvular dysfunction Vegetations/thickening with severe valvular dysfunction OR valve replacement	□ Yes □ No □ Yes □ No □ Yes □ No
	Pulmonary hypertension:	
	Right heart catheterization showing mean pulmonary artery pressure > 20 mm Hg or echocardiogram showing estimated pulmonary artery systolic pressure > 40 mm Hg Pulmonary hypertension with cor pulmonale	□ Yes □ No □ Yes □ No
	Pericarditis, pericardial thickening, or pericardiectomy	□ Yes □ No
	Carotid endarterectomy/stenting	□ Yes □ No
Vascular	Arterial thrombosis including organ infarcts other than MI and stroke	□ Yes □ No
	Peripheral ischemia/gangrene with tissue loss:	
	one site More than one site	□ Yes □ No □ Yes □ No
	Peripheral arterial disease	□ Yes □ No
	Post–venous thrombosis complications	□ Yes □ No
Pulmonary	Pulmonary fibrosis:	
	Mild or moderate pulmonary fibrosis Advanced pulmonary fibrosis	□ Yes □ No □ Yes □ No
	Pulmonary infarction or resection not for malignancy	□ Yes □ No
	Chronic dyspnoea	□ Yes □ No
Musculoskeletal	Avascular necrosis	
	one site More than one site	□ Yes □ No □ Yes □ No
	Osteoporosis with fracture or nontraumatic long bone fractures for children	□ Yes □ No
	Erosive arthropathy	□ Yes □ No
	Jaccoud arthropathy	□ Yes □ No
	Joint replacement	□ Yes □ No
	Tendon rupture	□ Yes □ No
Skin/mucocutaneous	Cutaneous scarring or atrophy (including lipoatrophy):	
	Affecting the trunk and/or extremities Affecting the head and/or neck (excluding the scalp)	□ Yes □ No □ Yes □ No
	Dyspigmentation	
	More than six mo but less than 12 mo duration More than 12 mo duration	□ Yes □ No □ Yes □ No
	Scarring chronic alopecia:	
	1 quadrant 2 quadrants 3 – 4 quadrants	□ Yes □ No □ Yes □ No □ Yes □ No
Malignancy	Malignancy	
	Nonmelanoma skin cancer Any other primary malignancy 2 or more malignancies	□ Yes □ No □ Yes □ No □ Yes □ No
Organ transplantation	Organ transplantation (nonkidney)	□ Yes □ No

* Items and subitems are presented in their relevant clinical domains. Items with subitems are defined to reflect current guidelines and/or clinical expert opinion. Items and subitems need to be present for a minimum of 6 mo unless otherwise stated in the glossary. Items occurring after the diagnosis of SLE DO NOT need to be directly attributed to SLE. Items occurring before the diagnosis of SLE should be scored if attributable to an SLE-related process† by the assessor. Only score the highest scoring subitem for each item.

† Examples of an “SLE-related process” before the diagnosis of SLE could include manifestations of antiphospholipid syndrome, interstitial lung disease due to a disease process that evolves to SLE, or deforming inflammatory arthritis that evolves to SLE.

EF, ejection fraction; eGFR, estimated glomerular filtration rate; MI, myocardial infarction; SLE, systemic lupus erythematosus.

### Item definitions and subitem proposals

The clinical domain groups were tasked with proposing definitions for each of the items, based on current evidence-based practice, specialty guidelines, and their clinical expertise. In developing precise definitions for each item, potential subitems based on severity for 17 items (43.6%) were proposed (table 3 and figure 1). For example, by expert consensus, ischaemic heart disease is subdivided as asymptomatic ischemia on testing, symptomatic angina with confirmatory tests, myocardial infarction (clinical presentation and confirmatory tests), and multiple myocardial infarctions and/or coronary artery bypass grafting. Similarly, kidney function impairment is subdivided according to Kidney Disease Improving Global Outcomes (KDIGO) categories of estimated glomerular filtration rate (eGFR).^15^ Chronicity findings in the kidney biopsy were also graded according to the classification of the International Society of Nephrology/Renal Pathology Society.^16^

## Discussion

Using a combined data-driven and expert/patient-based consensus approach, domains, items, and subitems that reflect the new damage construct in SLE have been developed consisting of 39 candidate items across 13 domains to be taken forward to the next stages. During the item generation phase, just over half of all items were nominated by both literature review and Delphi exercise. The Delphi exercise, which included a wide and diverse group of contributors, provided 103 unique items for further consideration. Our data confirm the value of large group exercises to maximise the scope of new items to consider for a revised index. Novel contributions to the development of the new damage index include the addition of paediatric growth items, use of prediagnosis SLE-related damage, and graded severity subitems.

Eleven individual items from the original SDI were excluded, including cranial neuropathy and proteinuria, or subsumed in newly defined items, including pleural fibrosis and skin ulceration. The neuropsychiatric clinical domain group recommended excluding cranial neuropathy, as this rare manifestation is usually reversible with therapy and more often reflects disease activity than irreversible damage.^17^ In the SLICC Inception Cohort, Hanly *et al* observed that over 50% of cranial neuropathies had resolved by 1 year. Time to resolution was also significantly shorter than for peripheral neuropathies, supporting the view that cranial neuropathies in SLE are typically manifestations of activity. Moreover, the prevalence of persistent cranial neuropathies that might reflect damage was well below 1%, so this item was excluded on the grounds of both reversibility and rarity.^18^ Similarly, multiple studies indicate that SLE psychosis is often transient and reversible and is strongly associated with disease activity. Paholpak *et al* found that 97.1% of psychotic episodes (36 episodes in 35 patients) fully remitted.^19^ Hanly *et al* reported that more than 80% of psychotic events had resolved by the second annual assessment following the onset of the event.^20^ Proteinuria, another excluded item, did not reach the threshold for inclusion with the item reduction committee. Its inclusion was also reconsidered and reviewed by the renal clinical domain group. Persistent proteinuria can reflect either inflammatory activity or chronic damage.^21^ When linked to chronic damage, it is frequently accompanied by other measures of kidney damages, such as reduced eGFR or chronicity features on biopsy, which are already captured in our item list. Furthermore, the growing long-term use of antiproteinuric therapy in patients with lupus nephritis may impact and cause fluctuations in the levels of proteinuria over time. For all the above reasons, the consensus was that proteinuria was excluded.

With the exception of surgical resection of bowel below the duodenum, spleen, liver, or gallbladder (not for malignancy) and motility disorders including oesophageal dysmotility and pseudo–bowel obstruction, most GI domain items were excluded as they were deemed infrequent (excluded for relevance) by the respective clinical domain group. The Spanish historical cohort study (RELESSER) reported GI damage in 3.7% of 3658 SLE cases, distributed as follows: infarction or resection involving the bowel, spleen, or liver (2.8%); pancreatic insufficiency (0.3%); stricture or upper GI surgery (0.3%); mesenteric insufficiency (0.2%); and chronic peritonitis (0.2%).^22^ Liver involvement and other GI manifestations are not included in the 2019 EULAR/ACR Classification Criteria for SLE.^10 13^ However, 25% to 50% of patients with SLE may experience transient liver disease, predominantly subclinical, with mild liver enzyme elevations seen in up to one-third of patients as part of active disease.^23 24^ Despite this, large-scale multicenter studies indicate that liver involvement is a rare cause of morbidity or mortality in patients with SLE.^25^

Haematology and reproduction/pregnancy were two newly proposed organ systems during the item generation phase. The item reduction committee excluded all reproduction/pregnancy items and half of the haematology items. Reproductive and pregnancy-related items were not deemed consistent with the current construct of damage. For example, pre-eclampsia and adverse pregnancy outcomes have been shown to be risk factors for future cardiovascular disease in women in the general population.^26^ In addition, congenital heart block, although proposed, does not affect the mother and so does not reflect the damage construct in the mother.

For haematology, items such as aplasia, myelodysplasia, or myelofibrosis due to SLE were excluded as they are rare events that may resolve with treatment. For instance, Chalayer *et al* studied 30 patients with bone marrow involvement from 19 centres. They reported that 24 episodes (including relapses) showed a treatment response, with a mean time to resolution of 9 months (range 1–75 months). Several cases of myelofibrosis in this cohort also demonstrated clinical improvement.^27^ Additionally, the haematology clinical domain group suggested removing splenectomy and splenic infarction to prevent double counting, as these overlapped with items in other organ systems, namely surgical resection of the spleen in the GI domain and arterial thrombosis in the vascular domain.

A novel feature from this phase is the clustering of items under domains and subdivision of some items into subitems. The clinical domain groups recommended clustering related items within each domain. This is similar to the recommendation given by an SLE expert panel in the development of the EULAR/ACR classification criteria, in which items are clustered if they are clinically or physiologically linked.^28^ For example, mild and major cognitive impairment items are separated only by the degree in severity. Similarly chronic kidney disease is defined in stages aligned to widely used international guidance. Furthermore, the clinical domain groups proposed subdividing items into subitems based on severity or grade. In some instances, the subitems are based on pre-existing internationally accepted categories (eg, KDIGO thresholds for eGFR,^15^ whereas others are based on expert clinical recommendations (neurocognitive impairment includes mild and major impairment). The ability to delineate subitems offers a more detailed and clinically relevant assessment of organ damage in patients with SLE. It reflects current evidence-based medical practice and is likely to improve sensitivity of the index in SLE populations. This improvement in sensitivity of measuring damage with SLE may result in an amelioration of the floor effect observed in the original SDI and result in improved sensitivity to change. For example, the threshold to score damage in the kidney now includes both chronicity on renal biopsy findings (not previously included) and an eGFR of 60% or less, which is a higher eGFR threshold than currently in the SDI (< 50%). For pulmonary fibrosis, the chest radiograph (current SDI) has a sensitivity of 63% compared with high resolution computerised tomography (included in our new definition), the current clinical criterion standard^29^; likewise in pulmonary hypertension, a loud P2 and/or parasternal heave (current SDI) have sensitivities of 30% to 40% compared with the gold standard of right heart catheterization (100% sensitivity) of the new proposed index.^30^

For each item, we also specifically asked the respective expert group to determine whether a timing of 6 months or more was an evidence-based way to reflect the construct of damage for that particular item. For most items, the 6 month duration was deemed a practical and plausible duration to distinguish activity from damage. For a small number of items, some of the definitions vary from the 6 month cut point. For example, ovarian insufficiency requires only a 3 month persistence, aligned with international guidance.^31 32^ Similarly, dyspigmentation has a 12 month threshold, reflecting expert opinion that the time reversibility of dyspigmentation can take up to 12 months, beyond which it is likely to be permanent.

Our study has several strengths. First, an inclusive sample of diverse individuals with respect to sex, age, race, clinical experience, and specialties ensures a broad representation of perspectives within both lupus research as well as patient communities. The heterogeneity in our sample population not only adds to the generalizability of study findings but also gives assurance to the insights drawn from the item generation and iterative reduction phases. Our process involved careful refinement across multiple rounds, providing high face validity in capturing a wide range of items covering damage accrual in SLE. In addition, the integration of patient input throughout the study—from study design to item evaluation—reflects a commitment to research that is patient-centric and ensures that this SDI revision is aligned with the needs and perceptions of those living with lupus.

We acknowledge several limitations of this study. First, the reliance on a nonsystematic literature review during the item generation phase may have introduced bias and potentially overlooked relevant literature. To minimise the risk of omitting relevant damage items, we increased the Delphi rounds from two to three. In the first and second rounds of the Delphi exercise, we included an open-ended question asking participants to nominate any items that potentially could be relevant for a revised SDI. Second, the underrepresentation of participants from low- and middle-income countries (LMICs) raises questions about the generalizability of the study findings to these regions. To address this gap, we purposely shared our preliminary item list with several LMICs, including countries in Africa. A key reflection from this was that a new index has the potential to support colleagues in LMIC settings to lobby policy makers and payers for better standards of care for their patients. So, although some diagnostics are not yet globally available, retaining these definitions will help raise standards of care for patients with SLE across LMIC settings. LMIC physicians will also be involved in later steps in the index development.

Our item generation and reduction phases have resulted in 39 candidate items to be taken forward to the next stages. The removal of outdated items, items now known to reflect inflammation, and the inclusion of new items, including paediatric items, better reflects the modern construct of damage in SLE across the life course. The inclusion of subitems based on severity or grade is novel. It reflects the evolution in current evidence-based medical practice over the past 30 years and so is likely to improve sensitivity of the index in SLE populations. The next phases will evaluate the item thresholds, how the weight of each item and subitem contributes to the concept of damage in SLE, and the validation of the revised SDI.

## Supplementary material

10.1136/lupus-2026-002282 online supplemental file 1

10.1136/lupus-2026-002282 online supplemental table 1

10.1136/lupus-2026-002282 online supplemental figure 1

10.1136/lupus-2026-002282 online supplemental figure 2

## References

[1] Fanouriakis A, Tziolos N, Bertsias G (2021). Update οn the diagnosis and management of systemic lupus erythematosus. Ann Rheum Dis.

[2] Tektonidou MG, Lewandowski LB, Hu J (2017). Survival in adults and children with systemic lupus erythematosus: a systematic review and Bayesian meta-analysis of studies from 1950 to 2016. Ann Rheum Dis.

[3] González LA, Alarcón GS (2017). The evolving concept of SLE comorbidities. Expert Rev Clin Immunol.

[4] Muñoz-Grajales C, Yilmaz EB, Svenungsson E (2023). Systemic lupus erythematosus and damage: What has changed over the past 20 years?. Best Pract Res Clin Rheumatol.

[5] Gladman D, Ginzler E, Goldsmith C (1996). The development and initial validation of the systemic lupus international collaborating clinics/American college of rheumatology damage index for systemic lupus erythematosus. Arthritis & Rheumatism.

[6] Sutton EJ, Davidson JE, Bruce IN (2013). The systemic lupus international collaborating clinics (SLICC) damage index: a systematic literature review. Semin Arthritis Rheum.

[7] Barber MRW, Johnson SR, Gladman DD (2021). Evolving concepts in systemic lupus erythematosus damage assessment. Nat Rev Rheumatol.

[8] Eder L, Urowitz MB, Gladman DD (2013). Damage in lupus patients--what have we learned so far?. Lupus.

[9] Johnson SR, Gladman DD, Brunner HI (2023). Evaluating the Construct of Damage in Systemic Lupus Erythematosus. Arthritis Care Res (Hoboken).

[10] Aringer M, Costenbader K, Daikh D (2019). 2019 European League Against Rheumatism/American College of Rheumatology Classification Criteria for Systemic Lupus Erythematosus. Arthritis & Rheumatology.

[11] Fransen J, Johnson SR, van den Hoogen F (2012). Items for developing revised classification criteria in systemic sclerosis: Results of a consensus exercise. Arthritis Care Res (Hoboken).

[12] van den Hoogen F, Khanna D, Fransen J (2013). 2013 Classification Criteria for Systemic Sclerosis: An American College of Rheumatology/European League Against Rheumatism Collaborative Initiative. Arthritis & Rheumatism.

[13] Schmajuk G, Hoyer BF, Aringer M (2018). Multicenter Delphi Exercise to Identify Important Key Items for Classifying Systemic Lupus Erythematosus. Arthritis Care Res (Hoboken).

[14] Dillman DA, Smyth JD, Christian LM (2011). Internet, Mail, and Mixed-Mode Surveys: The Tailored Design Method.

[15] Rovin BH, Ayoub IM, Chan TM (2024). KDIGO 2024 Clinical Practice Guideline for the management of LUPUS NEPHRITIS. Kidney Int.

[16] Bajema IM, Wilhelmus S, Alpers CE (2018). Revision of the International Society of Nephrology/Renal Pathology Society classification for lupus nephritis: clarification of definitions, and modified National Institutes of Health activity and chronicity indices. Kidney Int.

[17] Saleh Z, Menassa J, Abbas O (2010). Cranial nerve VI palsy as a rare initial presentation of systemic lupus erythematosus: case report and review of the literature. Lupus.

[18] Hanly JG, Li Q, Su L (2020). Peripheral Nervous System Disease in Systemic Lupus Erythematosus: Results From an International Inception Cohort Study. Arthritis & Rheumatology.

[19] Paholpak P, Rangseekajee P, Foocharoen C (2012). Characteristics, treatments and outcome of psychosis in Thai SLE patients. J Psychosom Res.

[20] Hanly JG, Li Q, Su L (2019). Psychosis in Systemic Lupus Erythematosus: Results From an International Inception Cohort Study. Arthritis & Rheumatology.

[21] Ergun MC, Aktas E, Sahin AT (2024). Systemic Immune-Inflammation Index as a Potential Biomarker for Assessing Disease Activity and Predicting Proteinuria Development in Systemic Lupus Erythematosus. Cureus.

[22] Tejera Segura B, Altabás González I, Rúa-Figueroa I (2021). Relevance of gastrointestinal manifestations in a large Spanish cohort of patients with systemic lupus erythematosus: what do we know?. Rheumatology (Oxford).

[23] Bessone F, Poles N, Roma MG (2014). Challenge of liver disease in systemic lupus erythematosus: Clues for diagnosis and hints for pathogenesis. World J Hepatol.

[24] van Hoek B (1996). The spectrum of liver disease in systemic lupus erythematosus. Neth J Med.

[25] Afzal W, Haghi M, Hasni SA (2020). Lupus hepatitis, more than just elevated liver enzymes. Scand J Rheumatol.

[26] Wu P, Haththotuwa R, Kwok CS (2017). Preeclampsia and Future Cardiovascular Health: A Systematic Review and Meta-Analysis. Circ Cardiovasc Qual Outcomes.

[27] Chalayer E, Costedoat-Chalumeau N, Beyne-Rauzy O (2017). Bone marrow involvement in systemic lupus erythematosus. QJM.

[28] Johnson SR, Khanna D, Daikh D (2019). Use of Consensus Methodology to Determine Candidate Items for Systemic Lupus Erythematosus Classification Criteria. J Rheumatol.

[29] Ghodrati S, Pugashetti JV, Kadoch MA (2022). Diagnostic Accuracy of Chest Radiography for Detecting Fibrotic Interstitial Lung Disease. Ann Am Thorac Soc.

[30] Braganza M, Shaw J, Solverson K (2019). A Prospective Evaluation of the Diagnostic Accuracy of the Physical Examination for Pulmonary Hypertension. Chest.

[31] American College of Obstetricians and Gynecologists (2014). Primary ovarian insufficiency in adolescents and young women. https://www.acog.org/clinical/clinical-guidance/committee-opinion/articles/2014/07/primary-ovarian-insufficiency-in-adolescents-and-young-women.

[32] Nelson LM (2009). Clinical practice. Primary ovarian insufficiency. N Engl J Med.

[33] Liang MH, Corzillius M, Bae SC (1999). The American College of Rheumatology nomenclature and case definitions for neuropsychiatric lupus syndromes. Arthritis & Rheumatism.

[34] Transverse Myelitis Consortium Working Group* (2002). Proposed diagnostic criteria and nosology of acute transverse myelitis. Neurology.

[35] Thompson AJ, Banwell BL, Barkhof F (2018). Diagnosis of multiple sclerosis: 2017 revisions of the McDonald criteria. Lancet Neurol.

[36] Wingerchuk DM, Banwell B, Bennett JL (2015). International consensus diagnostic criteria for neuromyelitis optica spectrum disorders. Neurology.

[37] American Psychiatric Association (2013). Diagnostic and Statistical Manual of Mental Disorders.

[38] de Andrade FA, Guimarães Moreira Balbi G, Bortoloti de Azevedo LG (2017). Neuro-ophthalmologic manifestations in systemic lupus erythematosus. Lupus.

[39] Fong KY, Thumboo J, Koh ET (1997). Systemic lupus erythematosus: initial manifestations and clinical features after 10 years of disease. Ann Acad Med Singap.

[40] Marmor MF, Kellner U, Lai TYY (2016). Recommendations on Screening for Chloroquine and Hydroxychloroquine Retinopathy (2016 Revision). Ophthalmology.

[41] American Diabetes Association (2014). Standards of Medical Care in Diabetes—2014. Diabetes Care.

[42] Sbardella E, Isidori AM, Woods CP (2017). Baseline morning cortisol level as a predictor of pituitary-adrenal reserve: a comparison across three assays. Clin Endocrinol (Oxf).

[43] Woods CP, Argese N, Chapman M (2015). Adrenal suppression in patients taking inhaled glucocorticoids is highly prevalent and management can be guided by morning cortisol. Eur J Endocrinol.

[44] Fragoso Perozo AFD, Fontes R, Lopes FP (2023). Morning serum cortisol role in the adrenal insufficiency diagnosis with modern cortisol assays. J Endocrinol Invest.

[45] Clark PM, Neylon I, Raggatt PR (1998). Defining the normal cortisol response to the short Synacthen test: implications for the investigation of hypothalamic-pituitary disorders. Clin Endocrinol (Oxf).

[46] Fleseriu M, Auchus R, Bancos I (2021). Consensus on diagnosis and management of Cushing’s disease: a guideline update. Lancet Diabetes Endocrinol.

[47] Joseph RM, Hunter AL, Ray DW (2016). Systemic glucocorticoid therapy and adrenal insufficiency in adults: A systematic review. Semin Arthritis Rheum.

[48] Sagar R, Mackie S, W Morgan A (2021). Evaluating tertiary adrenal insufficiency in rheumatology patients on long-term systemic glucocorticoid treatment. Clin Endocrinol (Oxf).

[49] Bhasin S, Brito JP, Cunningham GR (2018). Testosterone Therapy in Men With Hypogonadism: An Endocrine Society Clinical Practice Guideline. J Clin Endocrinol Metab.

[50] Ohri AJ, Shah CG, Udani AH (2022). Intestinal Pseudo-Obstruction - An Under-Recognized Presentation of Systemic Lupus Erythematosus. Indian J Nephrol.

[51] Heidenreich PA, Bozkurt B, Aguilar D (2022). 2022 AHA/ACC/HFSA Guideline for the Management of Heart Failure: A Report of the American College of Cardiology/American Heart Association Joint Committee on Clinical Practice Guidelines. J Am Coll Cardiol.

[52] Bai AD, Steinberg M, Showler A (2017). Diagnostic Accuracy of Transthoracic Echocardiography for Infective Endocarditis Findings Using Transesophageal Echocardiography as the Reference Standard: A Meta-Analysis. J Am Soc Echocardiogr.

[53] Maron BA (2023). Revised Definition of Pulmonary Hypertension and Approach to Management: A Clinical Primer. J Am Heart Assoc.

[54] Donohue CM, Adler JV, Bolton LL (2020). Peripheral arterial disease screening and diagnostic practice: A scoping review. Int Wound J.

[55] Flaherty KR, King TE, Raghu G (2004). Idiopathic interstitial pneumonia: what is the effect of a multidisciplinary approach to diagnosis?. Am J Respir Crit Care Med.

[56] Pellegrino R, Viegi G, Brusasco V (2005). Interpretative strategies for lung function tests. Eur Respir J.

[57] Raghu G, Remy-Jardin M, Richeldi L (2022). Idiopathic Pulmonary Fibrosis (an Update) and Progressive Pulmonary Fibrosis in Adults: An Official ATS/ERS/JRS/ALAT Clinical Practice Guideline. Am J Respir Crit Care Med.

[58] Pugashetti JV, Adegunsoye A, Wu Z (2023). Validation of Proposed Criteria for Progressive Pulmonary Fibrosis. Am J Respir Crit Care Med.

[59] Kaptein FHJ, Kroft LJM, Hammerschlag G (2021). Pulmonary infarction in acute pulmonary embolism. Thromb Res.

[60] Maher TM, Stowasser S, Voss F (2023). Decline in forced vital capacity as a surrogate for mortality in patients with pulmonary fibrosis. Respirology.

[61] Şenkal N, Kıyan E, Demir AA (2022). Interstitial lung disease in patients with systemic lupus erythematosus: a cohort study. Turk J Med Sci.

[62] Assouline-Dayan Y, Chang C, Greenspan A (2002). Pathogenesis and natural history of osteonecrosis. Semin Arthritis Rheum.

[63] Mont MA, Marulanda GA, Jones LC (2006). Systematic analysis of classification systems for osteonecrosis of the femoral head. J Bone Joint Surg Am.

[64] Seamon J, Keller T, Saleh J (2012). The pathogenesis of nontraumatic osteonecrosis. Arthritis.

[65] Eastell R, O’Neill TW, Hofbauer LC (2016). Postmenopausal osteoporosis. Nat Rev Dis Primers.

[66] Roux C, Gandjbakhch F, Pierreisnard A (2019). Ultrasonographic criteria for the diagnosis of erosive rheumatoid arthritis using osteoarthritic patients as controls compared to validated radiographic criteria. Joint Bone Spine.

[67] Schett G (2007). Erosive arthritis. Arthritis Res Ther.

[68] Santiago M (2020). Jaccoud-type lupus arthropathy: practical classification criteria. Lupus Sci Med.

[69] Santiago MB (2022). Jaccoud-type lupus arthropathy. Lupus.

[70] Chen C-H, Hsu C-W, Lu M-C (2019). Risk of joint replacement surgery in Taiwanese female adults with systemic lupus erythematosus: a population-based cohort study. BMC Musculoskelet Disord.

[71] Goodman SM, Springer BD, Chen AF (2022). 2022 American College of Rheumatology/American Association of Hip and Knee Surgeons Guideline for the Perioperative Management of Antirheumatic Medication in Patients With Rheumatic Diseases Undergoing Elective Total Hip or Total Knee Arthroplasty. J Arthroplasty.

[72] Furie RA, Chartash EK (1988). Tendon rupture in systemic lupus erythematosus. Semin Arthritis Rheum.

[73] Mak A (2014). Orthopedic surgery and its complication in systemic lupus erythematosus. World J Orthop.

[74] Albrecht J, Taylor L, Berlin JA (2005). The CLASI (Cutaneous Lupus Erythematosus Disease Area and Severity Index): an outcome instrument for cutaneous lupus erythematosus. J Invest Dermatol.

