# Supplementary material for: The lupus damage index revision program: Results from the item generation and reduction phases
Source: Lupus Sci Med. 2026 Aug 12;13(2):e002282. doi: 10.1136/lupus-2026-002282 (PMC13475098; doi:10.1136/lupus-2026-002282)
Supplement: Supplementary file 2 [file lupus-13-2-s002.pdf]

Supplementary Material

Table of contents

Supplementary Table 1. List of participants during item generation and item reduction phases of revised SDI development

Supplementary Table 2. Candidate items for the revised SDI from literature review and the first round Delphi exercise by organ system

Supplementary Table 3. Item reduction decisions by the Item Reduction Committee (n = 190 items)

Supplementary Table 4. Excluded items and exclusion reasons by the Clinical Domain Groups

Supplementary Table 1. List of participants during item generation and item reduction phases of revised Lupus Damage Index development (alphabetically ordered list by surnames)

SDI Revision Leadership Team

Ian N. Bruce, Megan R.W. Barber, Ann E. Clarke, Sindhu R. Johnson, Burak Kundakci

SDI Revision Literature Review Groups

Jiacai Cho, Nathalie Costedoat-Chalumeau, Hermine Brunner, Megan R.W. Barber, Ellen M. Ginzler, John G. Hanly, Abida Hasan, Colton Hoffer, Murat Inanc, Naureen Kabani, Alexandra Legge, Deborah Levy, Kaitlin Lima, Livia Lindoso, Anselm Mak, Rosalind Ramsey-Goldman, Noelle A. Rolle, Guillermo Ruiz-Irastorza, Clovis A. Silva, Farah Tamirou, Vitor C. Trindade, Évelyne Vinet

SDI Revision Delphi Panel

Alfredo Aguirre, Aysun Aksoy, Basma Mohammed Medhat Ali, Gelsomina Alle, Ibrahim Almaghlouth, Khalid A. Alnaqbi, Graciela S. Alarcón, Maame-Boatemaa Amissah-Arthur, Martin Aringer, Laurent Arnaud, Anca Askanase, Sang-Cheol Bae, Sasha Bernatsky, Eloisa Bonfa, Josiane Bourré-Tessier, Ian N. Bruce, Hermine Brunner, Armando A. Calvo Quiroz, Oswaldo Castaneda, François Chasset, Walter Winn Chatham, Kim Cheema, May Y. Choi, Ann E. Clarke, Fabrizio Conti, Nathalie Costedoat-Chalumeau, Adriana Danowski, Alvaro Danza, Dzifa Dey, Hui Jen Ding, Ali Duarte-García, Sergio Duran-Barragan, Doruk Erkan, Jessica English, Serena Fasano, Aurore Fifi-Mah, Cindy Hilary Flower, Paul R. Fortin, Ellen M. Ginzler, Luis Alonso González, Caroline Gordon, Kaien Gu, Gaelle Guettrot-Imbert, Latika Gupta, John Hanly, Julia Harris, Sabrina Hoa, Murat Inanc, Emilia Inoue Sato, Catherine Ivory, Judith James, Sindhu R. Johnson, Andreas Jönsen, Michelle Jung, Diane L. Kamen, Leila Khalili, Amanda King, Marisa Klein-Gitelman, Andrea Knight, Bianca Lang, Aisha Lateef, Juris Lazovskis, Valérie Leclair, Jisoo Lee, Shin-Seok Lee, Alexandra Legge, Dag Leonard, Deborah Levy, S. Sam Lim, Wambui Machua, Meggan Mackay, Anselm Mak, Daniel Martín-Iglesias, Loreto Massardo, Taneisha Khadine Mcghie, Maureen McMahon, Arielle Mendel, Eric Morand, Gabriella Moroni, Marta Mosca, Pintip Ngamjanyaporn, Ola Nived, Sean O'Neill, Pooja Patel, Christine A. Peschken, Michelle Petri, Bernardo Pons-Estel, Guillermo Pons-Estel, Gaafar Mohamed Ragab, Anisur Rahman, Rosalind Ramsey-Goldman, Simona Rednic, John Reynolds, Benjamin Rhodes, Ariadne Katina Riley, Noelle Rolle, Brad Rovin, Guillermo Ruiz-Irastorza, Jorge Sanchez-Guerrero, Syahrul Sazliyana Shaharir, Seung Cheol Shim, Earl Silverman, Christopher Sjöwall, Sarah Skeoch, Frida Strauss, Elisabet Svenungsson, Trudy Taylor, Maria Tektonidou, Julian Thumboo, Homa Timlin, Sofia Tosounidou, Zahi Touma, Manuel F. Ugarte-Gil, Murray Urowitz, Gloria Vasquez, Luis M. Vilá, Évelyne Vinet, Ronald Van Vollenhoven, Alexandre Voskuyl, Anne Voss, Daniel J. Wallace, Joshua Waytz, Victoria P. Werth, Jill Wong

The SDI Revision Item Reduction Committee

Laurent Arnaud, Megan R.W. Barber, Ian N. Bruce, Hermine Brunner, Ann E. Clarke, Dafna Gladman, Murat Inanc, Soren Jacobsen, Sindhu R. Johnson, Anselm Mak, Michelle Petri, Rosalind Ramsey-Goldman, Guillermo Ruiz-Irastorza, Alexandre Voskuyl

SDI Revision Clinical Domain Groups

Sulaiman Al-Mayouf, Simone Appenzeller, Cynthia Aranow, Bahar Artim-Esen, Anca Askanase, Tadej Avcin, Jo Blair, Hermine Brunner, Laura Bueno, Nathalie Costedoat-Chalumeau, Doruk Erkan, John D. Fisk, Ellen M. Ginzler, John G. Hanly, Colton Hoffer, Murat Inanc, Kenneth Kalunian, Marisa Klein-Gitelman, Alexandra Legge, Deborah Levy, Anselm Mak, Daniel Martín-Iglesias, Joseph F. Merola, Angela Migowa, Takako Miyamae, Gabriella Moroni, Marta Mosca, Gülfer Okumuş, Julie Park, Anisur Rahman, Noelle Rolle, Rosalind Ramsey-Goldman, Brad Rovin, Guillermo Ruiz-Irastorza, Lou Salciccioli, Davinder Singh-Grewal, Eve Smith, Sarah Swauger, Caitlin Tarlton, Frank Sen-Hee Tay, Victoria P. Werth, Samantha Yang, Yew Sen Yuen

Supplementary Table 2. Candidate items for the revised SDI from literature review and the first round Delphi exercise by organ system

Organ system	Literature review	Both exercises	1st round Delphi exercise
**Neuropsychiatric**		Autonomic disorder<br>Cognitive impairment<br>Cranial neuropathy<br>Demyelinating disorder including Multiple Sclerosis<br>Ischemic or haemorrhagic stroke<br>Mood disorder<br>Peripheral neuropathy<br>Psychosis<br>Seizure disorder<br>Transverse myelitis	Attempted suicide<br>Cognitive dyslexia<br>Delirium<br>Dementia<br>Encephalitis<br>Executive function disorder<br>Hearing impairment<br>Images changes captured by functional MRI or SPECT<br>Incapacitating headaches<br>Insomnia<br>Loss of sensation<br>Movement disorders (ataxia, tremors, chorea, hemiballismus)<br>Neurological sequelae (any) from neuropsychiatric SLE<br>Poor scholastic achievement<br>Radiographically proven brain atrophy<br>Tinnitus
**Ocular**		Acquired enophthalmos<br>Cataract<br>Chloroquine and hydroxychloroquine maculopathy	Keratoconjunctivitis sicca<br>Optic neuritis<br>Persistent uveitis or scleritis<br>Retinal depigmentation and functional loss

	Chronic angle closure, open angle glaucoma and steroid induced glaucoma<br><br>Cicatricial conjunctivitis or symblepharon<br><br>Cicatricial ectropion (from DLE)<br><br>Corneal scarring<br><br>Madarosis<br><br>Optic atrophy<br><br>Permanent uniocular or binocular partial or complete loss of vision<br><br>Proliferative retinopathy<br><br>Retinal necrosis and/or retinal detachment (choroidopathy, lupus retinopathy, serous retinopathy)<br><br>Scleral thinning (as a result of anterior scleritis)	
**Endocrine/ Metabolic**	Adrenal insufficiency<br><br>Anti-insulin receptor antibodies with recurrent hypoglycaemia<br><br>Arterial hypertension<br><br>Cushing's Syndrome<br><br>Diabetes mellitus<br><br>Erectile dysfunction<br><br>Hyperlipidaemia<br><br>Hypopituitarism<br><br>Hypothyroidism	Cachexia / Wasting<br><br>Chronic fatigue syndrome<br><br>Eating disorder<br><br>Facial and/or extremity swelling<br><br>Hyperthyroidism<br><br>Metabolic syndrome<br><br>Persistent weight gain<br><br>Steroid-induced diabetes

		Premature ovarian failure/primary ovarian insufficiency<br><br>Reduced ovarian reserve<br><br>Testicular dysfunction with reduced sperm count and quality/gonadal dysfunction	
**Growth and Development**		Delayed puberty<br><br>Growth failure<br><br>Reduced final height	Non-traumatic long bone fractures for paediatric population
**Reproduction/ Pregnancy**	Gestational diabetes	Cervical dysplasia<br><br>Hypertensive disorders of pregnancy (gestational hypertension, preeclampsia, and/or eclampsia)<br><br>Infertility<br><br>Intra-uterine growth restriction / Small for gestational age<br><br>Miscarriage / fetal loss<br><br>Placental abruption<br><br>Preterm birth<br><br>Stillbirth	Congenital heart block<br><br>Neonatal lupus in offsprings<br><br>Obstetric antiphospholipid syndrome
**Hematologic**	Keloid scar linked to lymph node sampling	Functional asplenia<br><br>Non-Hodgkin's lymphoma<br><br>Spleen infarction<br><br>Splenectomy (for immune thrombocytopenia (ITP))<br><br>Splenic atrophy	Antiphospholipid syndrome requiring long-term anticoagulation<br><br>Aplastic anaemia<br><br>Bone marrow failure (aplasia, myelodysplasia or myelofibrosis)<br><br>Chronic anaemia<br><br>Prolonged cytopenias

	Treatment-related myelodysplastic syndrome	Thrombocytopenia, leukopenia
**Gastrointestinal**	Bowel stricture<br>Chronic liver disease and cirrhosis including nodular regenerative hyperplasia<br>Chronic Peritonitis<br>Dysphagia<br>Gastroparesis<br>Malabsorption and Protein Losing Enteropathy<br>Mesenteric Insufficiency<br>Motility disorders including oesophageal dysmotility<br>Pancreatic insufficiency or need to use pancreatic enzyme replacement<br>Surgical resection of bowel below duodenum, spleen, liver or gallbladder<br>Upper gastrointestinal tract surgery	Bariatric surgery<br>Budd-Chiari syndrome<br>Cholecystitis<br>Chronic ascites<br>End stage liver disease<br>Gastrointestinal bleed<br>Mesenteric Adenitis<br>Myositis with dysphagia<br>Vasculitis involving the mesentery, oesophagus, stomach, peritoneum, gallbladder, pancreas, liver resulting in organ necrosis
**Renal**	Decreased eGFR<br>Proteinuria<br>Renal biopsy findings (Chronicity index >4, OR Interstitial Fibrosis >25%, OR Tubular atrophy >25%)	Biopsy proved renal microangiopathy due to antiphospholipid syndrome even without GFR<50%<br>Bladder dysfunction<br>Chronic kidney disease<br>Chronic sub-nephrotic proteinuria<br>End-stage renal disease<br>Haematuria<br>Haemorrhagic cystitis

		Nephritic syndrome<br>Renal calculi<br>Renal infarction<br>Renal tubular defect<br>Sterile pyuria
**Cardiovascular**	Accelerated and early atherosclerosis<br>Angina or coronary artery bypass<br>Cardiomyopathy (ventricular dysfunction)<br>Complete AV block<br>Myocardial fibrosis<br>Myocardial infarction ever<br>Pericarditis, pericardial thickening or pericardiectomy<br>QT prolongation<br>Valve regurgitation<br>Valve stenosis<br>Valve thickening<br>Valve vegetations	Aortitis<br>Arrythmias<br>Arterial hypertension<br>Baseline oxygen requirement<br>Cardiogenic shock not due to sepsis<br>Carotid endarterectomy/stenting<br>Conduction system disease<br>Diastolic dysfunction<br>Pulmonary hypertension as a cardiac event<br>Right ventricular dysfunction
**Peripheral vascular**	Claudication<br>Intracardiac thrombus<br>Minor tissue loss (pulp space)<br>Significant tissue loss ever (e.g. loss of digit or limb)	Renal artery stenosis<br>Severe Raynaud’s<br>Thrombosis any (arterial or venous)

		Venous thrombosis or venous stasis	
**Pulmonary**	Respiratory insufficiency	Obstructive airway disease<br><br>Pleural fibrosis, pleurodesis or pleurectomy for refractory effusions<br><br>Pulmonary fibrosis<br><br>Pulmonary hypertension<br><br>Pulmonary infarction or resection not for malignancy<br><br>Shrinking lung	Bronchiectasis<br><br>Chronic diffuse alveolar haemorrhage<br><br>Diminished lung function (decreased DLCO, FEV1, FVC)<br><br>Lobectomy<br><br>Obstructive Sleep apnoea<br><br>Persistent decreased exercise tolerance/DLCO due to ILD or pulmonary emboli<br><br>Restrictive lung disease (based on PFT abnormalities)
**Musculoskeletal**		Atrophy or weakness<br><br>Avascular necrosis / Legg-Calve-Perthes disease / Perthes disease<br><br>Erosive arthropathy<br><br>Fibromyalgia<br><br>Fragility fractures<br><br>Frailty<br><br>Jaccoud's arthropathy<br><br>Muscle atrophy<br><br>Osteomalacia<br><br>Osteomyelitis<br><br>Osteoporosis with fracture<br><br>Osteosclerosis<br><br>Sarcopenia	Chronic gout with erosion and tophi<br><br>Inflammatory joint pain<br><br>Joint replacement<br><br>Osteoporosis even without fracture<br><br>Septic arthritis

		Tendon rupture	
**Skin/ Mucocutaneous**	Telangiectasia	Cicatricial skin atrophy<br>Dyspigmentation (hyperpigmentation or hypopigmentation)<br>Extensive striae<br>Panniculitis<br>Periodontal disease with tooth loss<br>Scarring (cutaneous)<br>Scarring chronic alopecia<br>Skin ulceration (excluding thrombosis) for > months	Calcinosis<br>Chronic discoid or subacute lesions<br>Easy bruisability<br>Hirsutism<br>Nasal septum perforation<br>Onychodystrophy<br>Persistent malar rash<br>Post venous thrombosis complications<br>Severe livedo reticularis<br>Severe photosensitivity limiting activities of daily living<br>Sjogren's syndrome<br>Xerostomia
**Others**		Malignancy	Carcinoma in situ (any site)<br>Organ transplantation (non-kidney)<br>Persistent hypogammaglobulinemia<br>Poverty<br>Severe or recurrent infections<br>Social isolation<br>Treatment related damage<br>Work disability

Supplementary Table 3. Item reduction decisions by the Item Reduction Committee (n = 190 items)*

Items	Inc/Excl	Inc/Excl	Inc/Excl	Inc/Excl	Inc/Excl	Inc/Excl	Inc/Excl	Inc/Excl	Inc/Excl	Inc/Excl	Inc/Excl	Inc/Excl	Inc/Excl	Inc/Excl
**NEUROPSYCHIATRIC**														
Ischemic or haemorrhagic stroke	Include	Include	Include	Include	Include	Include	Include	Include	Include	Include	Include	Include	Include	Include
Transverse myelitis	Include	Include	Include	Include	Include	Include	Include	Include	Include	Include	Include	Include	Include	Exclude
Cognitive impairment	Include	Exclude	Include	Include	Include	Include	Include	Include	Exclude	Include	Include	Include	Include	Include
Cranial neuropathy	Include	Include	Include	Include	Include	Include	Exclude	Include	Include	Include	Include	Include	Include	Exclude
Neurological sequelae (any) from neuropsychiatric SLE	Exclude	Exclude	Exclude	Include	Include	Include	Exclude	Exclude	Exclude	Exclude	Include	Include	Exclude	Include
Peripheral neuropathy	Exclude	Include	Include	Include	Include	Include	Include	Include	Exclude	Include	Include	Include	Exclude	Include
Seizure disorder	Include	Include	Include	Include	Include	Include	Include	Include	Include	Include	Include	Include	Include	Include
Dementia	Exclude	Include	Include	Include	Include	Exclude	Exclude	Exclude	Include	Exclude	Exclude	Include	Exclude	Exclude
Demyelinating disorder including Multiple Sclerosis	Include	Include	Include	Include	Include	Include	Exclude	Exclude	Include	Exclude	Include	Include	Exclude	Exclude
Movement disorders (Ataxia, tremors, chorea, hemiballismus)	Include	Include	Include	Include	Include	Include	Exclude	Include	Include	Include	Include	Include	Include	Include
Psychosis	Include	Exclude	Include	Include	Include	Include	Include	Include	Include	Include	Include	Include	Exclude	Exclude
Executive function disorder	Exclude	Exclude	Exclude	Exclude	Include	Include	Exclude	Exclude	Exclude	Exclude	Exclude	Include	Exclude	Exclude
Radiographically proven brain atrophy	Exclude	Exclude	Include	Exclude	Include	Include	Exclude	Exclude	Exclude	Exclude	Exclude	Include	Exclude	Include
Autonomic disorder	Exclude	Include	Include	Include	Exclude	Include	Exclude	Exclude	Exclude	Include	Include	Include	Exclude	Exclude
Cognitive dyslexia	Exclude	Exclude	Exclude	Exclude	Exclude	Exclude	Exclude	Exclude	Exclude	Exclude	Exclude	Include	Exclude	Exclude
Encephalitis	Exclude	Exclude	Exclude	Exclude	Include	Exclude	Exclude	Exclude	Exclude	Exclude	Exclude	Exclude	Exclude	Exclude
Image changes captured by functional MRI or SPECT	Exclude	Exclude	Exclude	Exclude	Exclude	Include	Exclude	Exclude	Exclude	Exclude	Exclude	Exclude	Exclude	Exclude
Incapacitating headaches	Exclude	Exclude	Include	Exclude	Exclude	Exclude	Exclude	Exclude	Exclude	Exclude	Exclude	Exclude	Exclude	Exclude
Intracranial hypertension	Include	Include	Include	Include	Exclude	Exclude	Include	Exclude	Include	Exclude	Exclude	Include	Include	Exclude
Hearing impairment	Include	Include	Include	Include	Exclude	Exclude	Exclude	Exclude	Exclude	Include	Exclude	Include	Exclude	Exclude
Loss of sensation	Exclude	Exclude	Exclude	Include	Exclude	Exclude	Exclude	Exclude	Exclude	Exclude	Exclude	Include	Exclude	Exclude
Delirium	Exclude	Exclude	Exclude	Exclude	Exclude	Exclude	Exclude	Exclude	Exclude	Exclude	Exclude	Exclude	Exclude	Exclude
Mood disorder	Exclude	Exclude	Exclude	Exclude	Exclude	Exclude	Exclude	Exclude	Exclude	Exclude	Exclude	Exclude	Exclude	Exclude
Attempted suicide	Exclude	Exclude	Exclude	Exclude	Exclude	Exclude	Exclude	Exclude	Exclude	Exclude	Exclude	Exclude	Exclude	Exclude
Insomnia	Exclude	Exclude	Exclude	Exclude	Exclude	Exclude	Exclude	Exclude	Exclude	Exclude	Exclude	Exclude	Exclude	Exclude
Poor scholastic achievement	Exclude	Exclude	Exclude	Exclude	Exclude	Exclude	Exclude	Exclude	Exclude	Exclude	Exclude	Exclude	Exclude	Exclude
Tinnitus	Exclude	Exclude	Exclude	Exclude	Exclude	Exclude	Exclude	Exclude	Exclude	Exclude	Exclude	Exclude	Exclude	Exclude

Items	Inc/Excl	Inc/Excl	Inc/Excl	Inc/Excl	Inc/Excl	Inc/Excl	Inc/Excl	Inc/Excl	Inc/Excl	Inc/Excl	Inc/Excl	Inc/Excl	Inc/Excl	Inc/Excl	I
**OCULAR**															
Chloroquine and hydroxychloroquine maculopathy	Exclude	Include	Include	Include	Include	Include	Include	Include	Include	Include	Include	Include	Include	Include	
Cataract	Include	Include	Include	Include	Include	Include	Include	Include	Include	Include	Include	Include	Include	Include	
Optic atrophy	Exclude	Include	Include	Include	Include	Include	Exclude	Include	Include	Include	Include	Include	Include	Include	
Permanent uniocular or binocular partial or complete loss of vision	Include	Include	Include	Include	Include	Include	Exclude	Include	Include	Include	Include	Include	Exclude	Include	
Retinal necrosis and/or retinal detachment	Exclude	Include	Include	Include	Include	Include	Exclude	Include	Include	Exclude	Include	Include	Exclude	Include	
Corneal scarring/Corneal ulceration	Include	Include	Include	Include	Include	Include	Exclude	Exclude	Include	Exclude	Include	Include	Exclude	Include	
Proliferative retinopathy	Exclude	Exclude	Exclude	Exclude	Exclude	Include	Exclude	Exclude	Include	Exclude	Include	Include	Exclude	Include	
Scleral thinning (as a result of anterior scleritis)	Exclude	Include	Exclude	Include	Include	Include	Exclude	Exclude	Include	Exclude	Include	Exclude	Exclude	Include	
Chronic angle closure, Open angle glaucoma and steroid induced glaucoma	Include	Include	Include	Include	Include	Include	Include	Exclude	Include	Exclude	Include	Include	Include	Include	
Cicatricial ectropion (from Discoid lupus erythematosus (DLE))	Exclude	Exclude	Include	Include	Exclude	Include	Exclude	Exclude	Exclude	Exclude	Include	Exclude	Exclude	Exclude	
Madarosis (from DLE)	Exclude	Exclude	Exclude	Include	Exclude	Include	Exclude	Exclude	Exclude	Exclude	Include	Exclude	Exclude	Exclude	
Optic neuropathy (due to ischemia or inflammation)	Exclude	Exclude	Exclude	Include	Include	Include	Exclude	Exclude	Include	Exclude	Include	Exclude	Exclude	Include	
Persistent uveitis or scleritis	Exclude	Include	Include	Exclude	Include	Include	Exclude	Exclude	Include	Exclude	Include	Include	Exclude	Exclude	
Retinal depigmentation and functional loss	Exclude	Exclude	Exclude	Exclude	Include	Include	Exclude	Exclude	Exclude	Exclude	Exclude	Include	Include	Exclude	
Cicatricial conjunctivitis or symblepharon	Exclude	exclude	Include	Include	Exclude	Include	Exclude	Exclude	Exclude	Exclude	Exclude	Include	Exclude	Exclude	
Keratoconjunctivitis sicca	Exclude	Include	Exclude	Include	Include	Include	Exclude	Include	Exclude	Exclude	Exclude	Include	Include	Exclude	
Acquired enophthalmos	Exclude	Exclude	Exclude	Exclude	Exclude	Exclude	Exclude	Exclude	Exclude	Exclude	Exclude	Exclude	Exclude	Exclude	Y

Items	Inc/Excl	Inc/Excl	Inc/Excl	Inc/Excl	Inc/Excl	Inc/Excl	Inc/Excl	Inc/Excl	Inc/Excl	Inc/Excl	Inc/Excl	Inc/Excl	Inc/Excl	Inc/Excl
**ENDOCRINE/METABOLIC**														
Premature ovarian failure/primary ovarian insufficiency	Include	Include	Include	Include	Include	Include	Include	Include	Include	Include	Include	Include	Include	Include
Steroid-induced diabetes	Exclude	Exclude	Exclude	Include	Include	Include	Include	Exclude	Include	Exclude	Include	Include	Include	Exclude
Adrenal insufficiency	Exclude	Include	Include	Include	Include	Include	Include	Include	Include	Exclude	Include	Include	Exclude	Include
Diabetes mellitus	Include	Include	Include	Include	Exclude	Include	Exclude	Include	Include	Include	Include	Include	Include	Include
Arterial hypertension	Exclude	Include	Include	Include	Exclude	Exclude	Exclude	Exclude	Exclude	Exclude	Include	Include	Exclude	Include
Cushing’s Syndrome	Exclude	Include	Include	Include	Include	Include	Include	Exclude	Exclude	Exclude	Exclude	Include	Exclude	Include
Metabolic syndrome	Exclude	Include	Exclude	Exclude	Exclude	Include	Exclude	Exclude	Include	Exclude	Exclude	Exclude	Exclude	Include
Reduced ovarian reserve	Exclude	Exclude	Exclude	Exclude	Exclude	Include	Exclude	Exclude	Exclude	Exclude	Exclude	Exclude	Include	Exclude
Testicular dysfunction with reduced sperm count and quality/gonadal dysfunction	Exclude	Exclude	Include	Include	Include	Include	Include	Exclude	Include	Exclude	Include	Include	Include	Include
Cachexia/Wastening	Exclude	Include	Include	Exclude	Exclude	Exclude	Exclude	Exclude	Exclude	Exclude	Exclude	Include	Exclude	Exclude
Chronic fatigue syndrome	Exclude	Exclude	Exclude	Include	Exclude	Include	Exclude	Exclude	Exclude	Exclude	Exclude	Exclude	Exclude	Exclude
Erectile dysfunction	Exclude	Exclude	Include	Include	Exclude	Include	Exclude	Exclude	Include	Exclude	Exclude	Include	Exclude	Exclude
Hyperlipidemia	Exclude	Include	Include	Exclude	Exclude	Exclude	Exclude	Exclude	Exclude	Exclude	Exclude	Exclude	Exclude	Exclude
Hypopituitarism	Exclude	Include	Include	Exclude	Include	Include	Exclude	Exclude	Include	Exclude	Exclude	Include	Exclude	Exclude
Persistent weight gain	Exclude	Exclude	Exclude	Exclude	Exclude	Exclude	Exclude	Exclude	Exclude	Exclude	Exclude	Exclude	Exclude	Exclude
Anti-insulin receptor antibodies with recurrent hypoglycaemia	Exclude	Exclude	Exclude	Exclude	Exclude	Exclude	Exclude	Exclude	Exclude	Exclude	Exclude	Exclude	Exclude	Exclude
Eating disorder	Exclude	Exclude	Exclude	Exclude	Exclude	Exclude	Exclude	Exclude	Exclude	Exclude	Exclude	Exclude	Exclude	Exclude
Hyperthyroidism	Exclude	Exclude	Exclude	Exclude	Exclude	Exclude	Exclude	Exclude	Exclude	Exclude	Exclude	Exclude	Exclude	Exclude
Hypothyroidism	Exclude	Exclude	Exclude	Exclude	Exclude	Exclude	Exclude	Exclude	Exclude	Exclude	Exclude	Exclude	Exclude	Exclude
Facial and/or extremity swelling	Exclude	Exclude	Exclude	Exclude	Exclude	Exclude	Exclude	Exclude	Exclude	Exclude	Exclude	Exclude	Exclude	Exclude
**GROWTH AND DEVELOPMENT**														
Growth failure	Include	Include	Include	Include	Include	Include	Include	Include	Include	Include	Include	Include	Exclude	Include
Non-traumatic long bone fractures for children	Exclude	Include	Include	Include	Include	Include	Exclude	Exclude	Include	Exclude	Include	Include	Exclude	Include
Delayed puberty	Include	Include	Include	Include	Include	Include	Include	Exclude	Include	Include	Include	Exclude	Exclude	Include
Reduced final height	Exclude	Include	Include	Exclude	Exclude	Include	Exclude	Exclude	Include	Include	Include	Exclude	Include	Include
Disordered puberty sequences	Exclude	Include	Include	Exclude	Exclude	Include	Exclude	Exclude	Include	Exclude	Exclude	Exclude	Exclude	Include
Limb leg discrepancies	Exclude	Exclude	Include	Exclude	Exclude	Exclude	Exclude	Exclude	Exclude	Exclude	Exclude	Exclude	Exclude	Include

Items	Inc/Excl	Inc/Excl	Inc/Excl	Inc/Excl	Inc/Excl	Inc/Excl	Inc/Excl	Inc/Excl	Inc/Excl	Inc/Excl	Inc/Excl	Inc/Excl	Inc/Excl	Inc/Excl
**REPRODUCTION/PREGNANCY**														
Congenital Heart Block	Exclude	Exclude	Exclude	Exclude	Include	Include	Include	Exclude	Exclude	Exclude	Exclude	Exclude	Exclude	Exclude
Hypertensive disorders of pregnancy	Exclude	Include	Include	Exclude	Exclude	Include	Include	Exclude	Exclude	Exclude	Exclude	Exclude	Exclude	Include
Infertility	Exclude	Exclude	Exclude	Exclude	Include	Include	Include	Exclude	Exclude	Exclude	Exclude	Include	Include	Exclude
Intra-uterine growth restriction / Small for gestational age	Exclude	Include	Exclude	Exclude	Exclude	Include	Exclude	Exclude	Exclude	Exclude	Exclude	Exclude	Exclude	Exclude
Miscarriage / fetal loss	Exclude	Include	Exclude	Exclude	Exclude	Include	Exclude	Exclude	Exclude	Exclude	Exclude	Exclude	Include	Exclude
Obstetric antiphospholipid syndrome	Exclude	Exclude	Exclude	Exclude	Exclude	Include	Exclude	Exclude	Exclude	Exclude	Exclude	Exclude	Exclude	Exclude
Preterm birth	Exclude	Include	Exclude	Exclude	Exclude	Include	Exclude	Exclude	Exclude	Exclude	Exclude	Exclude	Exclude	Exclude
Stillbirth	Exclude	Include	Exclude	Exclude	Exclude	Include	Exclude	Exclude	Exclude	Exclude	Exclude	Exclude	Include	Exclude
Cervical dysplasia	Exclude	Exclude	Exclude	Include	Exclude	Exclude	Exclude	Exclude	Include	Exclude	Exclude	Exclude	Exclude	Exclude
Gestational diabetes	Exclude	Exclude	Exclude	Exclude	Exclude	Include	Exclude	Exclude	Exclude	Exclude	Exclude	Exclude	Exclude	Exclude
Low anti-müllerian hormone levels	Exclude	Exclude	Exclude	Exclude	Exclude	Include	Exclude	Exclude	Exclude	Exclude	Exclude	Exclude	Exclude	Exclude
Neonatal lupus in offspring	Exclude	Exclude	Exclude	Exclude	Exclude	Exclude	Exclude	Exclude	Exclude	Exclude	Exclude	Exclude	Exclude	Exclude
Placental abruption	Exclude	Exclude	Exclude	Exclude	Exclude	Exclude	Exclude	Include	Exclude	Exclude	Exclude	Exclude	Exclude	Exclude
**HEMATOLOGIC**														
Splenectomy (for immune thrombocytopenia (ITP))	Include	Include	Exclude	Include	Include	Include	Include	Include	Include	Include	Include	Include	Exclude	Include
Aplastic anaemia	Exclude	Exclude	Exclude	Exclude	Include	Include	Exclude	Exclude	Include	Exclude	Include	Include	Exclude	Exclude
Bone marrow failure (aplasia, myelodysplasia or myelofibrosis)	Include	Include	Include	Include	Exclude	Include	Include	Exclude	Include	Include	Include	Include	Include	Exclude
Non-Hodgkin's lymphoma	Exclude	Include	Exclude	Include	Include	Include	Exclude	Exclude	Include	Exclude	Include	Include	Exclude	Exclude
Spleen infarction	Exclude	Exclude	Include	Exclude	Include	Include	Exclude	Include	Include	Exclude	Include	Include	Include	Include
Treatment-related myelodysplastic syndrome	Exclude	Include	Exclude	Include	Include	Include	Exclude	Exclude	Include	Exclude	Exclude	Include	Exclude	Exclude
Antiphospholipid syndrome	Exclude	Include	Include	Exclude	Exclude	Include	Exclude	Exclude	Exclude	Exclude	Exclude	Exclude	Exclude	Exclude
Functional asplenia	Exclude	Exclude	Include	Exclude	Include	Include	Exclude	Exclude	Include	Exclude	Exclude	Include	Exclude	Exclude
Prolonged cytopenias	Exclude	Exclude	Include	Exclude	Exclude	Include	Exclude	Exclude	Exclude	Exclude	Exclude	Exclude	Exclude	Exclude
Splenic atrophy	Exclude	Exclude	Exclude	Exclude	Exclude	Include	Exclude	Exclude	Include	Exclude	Exclude	Include	Include	Include
Chronic anaemia	Exclude	Exclude	Exclude	Exclude	Exclude	Exclude	Exclude	Exclude	Exclude	Exclude	Exclude	Exclude	Exclude	Exclude
Thrombocytopenia, leucopenia	Exclude	Exclude	Exclude	Exclude	Exclude	Exclude	Exclude	Exclude	Exclude	Exclude	Exclude	Exclude	Exclude	Exclude
Keloid scar linked to lymph node sampling	Exclude	Exclude	Exclude	Exclude	Exclude	Exclude	Exclude	Exclude	Exclude	Exclude	Exclude	Exclude	Exclude	Exclude

Items	Inc/Excl	Inc/Excl	Inc/Excl	Inc/Excl	Inc/Excl	Inc/Excl	Inc/Excl	Inc/Excl	Inc/Excl	Inc/Excl	Inc/Excl	Inc/Excl	Inc/Excl	Inc/Excl
**GASTROINTESTINAL**														
Bowel stricture	Exclude	Include	Include	Include	Include	Include	Exclude	Exclude	Include	Exclude	Include	Include	Exclude	Include
Budd-Chiari syndrome	Exclude	Include	Include	Include	Exclude	Include	Exclude	Exclude	Include	Exclude	Exclude	Include	Exclude	Include
Chronic ascites	Include	Exclude	Include	Include	Exclude	Include	Include	Exclude	Include	Exclude	Exclude	Include	Exclude	Exclude
Chronic liver disease and cirrhosis including nodular regenerative hyperplasia	Include	Exclude	Include	Include	Exclude	Include	Exclude	Exclude	Include	Include	Include	Include	Include	Include
Chronic Peritonitis	Exclude	Include	Include	Include	Include	Include	Include	Exclude	Include	Exclude	Exclude	Include	Exclude	Exclude
End stage liver disease	Exclude	Include	Exclude	Exclude	Exclude	Include	Exclude	Exclude	Include	Exclude	Include	Include	Include	Include
Malabsorption and Protein Losing Enteropathy	Include	Include	Include	Include	Include	Exclude	Include	Exclude	Include	Exclude	Exclude	Include	Exclude	Include
Mesenteric Insufficiency	Include	Include	Include	Include	Exclude	Include	Exclude	Exclude	Include	Exclude	Exclude	Include	Exclude	Include
Pancreatic insufficiency or need to use pancreatic enzyme replacement	Exclude	Include	Include	Include	Include	Include	Include	Exclude	Include	Exclude	Include	Exclude	Include	Include
Surgical resection of bowel below the duodenum, spleen, liver or gallbladder	Include	Include	Include	Include	Exclude	Include	Include	Exclude	Include	Include	Include	Include	Include	Include
Vasculitis involving the mesentery, oesophagus, stomach, peritoneum, gallbladder, pancreas, liver resulting in organ necrosis	Exclude	Include	Include	Exclude	Exclude	Include	Exclude	Exclude	Include	Exclude	Exclude	Include	Exclude	Include
Gastroparesis	Exclude	Exclude	Exclude	Exclude	Exclude	Include	Exclude	Include	Include	Exclude	Exclude	Include	Exclude	Include
Motility disorders including oesophageal dysmotility and pseudo bowel obstruction	Include	Include	Include	Include	Include	Include	Exclude	Exclude	Include	Exclude	Exclude	Include	Exclude	Include
Dysphagia	Exclude	Include	Exclude	Exclude	Exclude	Exclude	Exclude	Exclude	Include	Exclude	Exclude	Include	Exclude	Exclude
Myositis with dysphagia	Exclude	Exclude	Exclude	Exclude	Exclude	Exclude	Exclude	Exclude	Exclude	Exclude	Exclude	Include	Exclude	Exclude
Upper gastrointestinal tract surgery	Exclude	Exclude	Exclude	Include	Exclude	Exclude	Exclude	Exclude	Exclude	Exclude	Exclude	Include	Exclude	Exclude
Cholecystitis	Exclude	Exclude	Exclude	Exclude	Exclude	Exclude	Exclude	Exclude	Exclude	Exclude	Exclude	Exclude	Exclude	Exclude
Gastrointestinal bleed	Exclude	Exclude	Exclude	Exclude	Exclude	Exclude	Exclude	Exclude	Exclude	Exclude	Exclude	Exclude	Exclude	Exclude
Mesenteric Adenitis	Exclude	Exclude	Exclude	Exclude	Exclude	Exclude	Exclude	Exclude	Exclude	Exclude	Exclude	Exclude	Exclude	Exclude
Bariatric surgery	Exclude	Exclude	Exclude	Exclude	Exclude	Exclude	Exclude	Include	Exclude	Exclude	Exclude	Exclude	Exclude	Exclude
**RENAL**														
Chronic kidney disease	Include	Include	Include	Include	Include	Include	Exclude	Include	Include	Exclude	Exclude	Include	Include	Include
Decreased eGFR	Exclude	Exclude	Exclude	Exclude	Exclude	Exclude	Include	Exclude	Exclude	Include	Include	Include	Include	Include
End-stage renal disease	Exclude	Exclude	Exclude	Exclude	Exclude	Include	Include	Exclude	Include	Include	Include	Include	Include	Include
Renal biopsy findings (Chronicity index >4, OR Interstitial Fibrosis >25%, OR Tubular atrophy >25%)	Exclude	Include	Include	Include	Include	Exclude	Exclude	Exclude	Include	Include	Include	Include	Exclude	Include
Biopsy proven renal microangiopathy due to antiphospholipid syndrome even without GFR less than 50%	Exclude	Include	Include	Include	Exclude	Include	Exclude	Exclude	Include	Exclude	Exclude	Include	Exclude	Include
Chronic sub-nephrotic proteinuria	Exclude	Exclude	Exclude	Include	Include	Include	Exclude	Exclude	Exclude	Exclude	Include	Include	Exclude	Include
Proteinuria	Include	Include	Include	Include	Include	Exclude	Exclude	Exclude	Include	Exclude	Include	Exclude	Include	Include
Renal infarction	Exclude	Include	Include	Include	Include	Include	Exclude	Exclude	Include	Exclude	Include	Include	Exclude	Include
Renal tubular defect	Include	Include	Include	Include	Exclude	Include	Exclude	Exclude	Include	Exclude	Exclude	Include	Exclude	Include
Bladder dysfunction	Exclude	Include	Exclude	Include	Exclude	Include	Exclude	Exclude	Include	Exclude	Exclude	Include	Exclude	Exclude
Haemorrhagic cystitis	Exclude	Exclude	Include	Include	Include	Exclude	Exclude	Exclude	Include	Exclude	Exclude	Exclude	Exclude	Exclude
Nephritic syndrome	Exclude	Exclude	Exclude	Exclude	Exclude	Exclude	Exclude	Exclude	Exclude	Exclude	Exclude	Exclude	Exclude	Exclude
Haematuria	Exclude	Exclude	Exclude	Exclude	Exclude	Exclude	Exclude	Exclude	Exclude	Exclude	Exclude	Exclude	Exclude	Exclude
Renal calculi	Exclude	Exclude	Exclude	Exclude	Exclude	Exclude	Exclude	Exclude	Exclude	Exclude	Exclude	Exclude	Exclude	Exclude
Sterile pyuria	Exclude	Exclude	Exclude	Exclude	Exclude	Exclude	Exclude	Include	Exclude	Exclude	Exclude	Exclude	Exclude	Exclude

Items	Inc/Excl	Inc/Excl	Inc/Excl	Inc/Excl	Inc/Excl	Inc/Excl	Inc/Excl	Inc/Excl	Inc/Excl	Inc/Excl	Inc/Excl	Inc/Excl	Inc/Excl	Inc/Excl
**CARDIOVASCULAR**														
Cardiomyopathy (ventricular dysfunction)	Include	Include	Include	Include	Include	Include	Include	Include	Include	Include	Include	Include	Exclude	Include
Myocardial infarction ever	Include	Include	Include	Include	Include	Include	Exclude	Include	Include	Include	Include	Include	Include	Include
Accelerated and early atherosclerosis	Exclude	Include	Include	Include	Exclude	Include	Include	Exclude	Exclude	Exclude	Include	Include	Exclude	Include
Angina or coronary artery bypass	Exclude	Exclude	Include	Include	Include	Include	Exclude	Include	Include	Include	Include	Include	Include	Include
Pericarditis, pericardial thickening or pericardiectomy	Include	Include	Include	Exclude	Include	Include	Include	Exclude	Include	Include	Include	Include	Exclude	Exclude
Pulmonary hypertension	Exclude	Include	Include	Include	Include	Include	Include	Exclude	Include	Include	Include	Include	Exclude	Exclude
Valve vegetations	Exclude	Exclude	Include	Include	Include	Include	Exclude	Exclude	Include	Exclude	Include	Include	Exclude	Include
Carotid endarterectomy/stenting	Exclude	Include	Include	Include	Include	Include	Exclude	Exclude	Include	Include	Include	Include	Exclude	Exclude
Complete AV block	Exclude	Exclude	Exclude	Include	Exclude	Exclude	Exclude	Exclude	Include	Include	Include	Include	Exclude	Exclude
Conduction system disease	Exclude	Include	Include	Include	Include	Include	Exclude	Exclude	Exclude	Exclude	Exclude	Include	Include	Exclude
Myocardial fibrosis	Exclude	Include	Include	Include	Exclude	Include	Exclude	Exclude	Include	Exclude	Include	Include	Exclude	Exclude
Right ventricular dysfunction	Exclude	Exclude	Exclude	Include	Include	Exclude	Exclude	Exclude	Exclude	Exclude	Exclude	Include	Exclude	Exclude
Valve stenosis	Include	Exclude	Include	Include	Include	Include	Exclude	Exclude	Include	Include	Exclude	Include	Include	Include
Valve thickening	Exclude	Exclude	Exclude	Exclude	Exclude	Exclude	Exclude	Exclude	Exclude	Exclude	Exclude	Include	Exclude	Include
Arrhythmias	Include	Include	Exclude	Include	Exclude	Include	Exclude	Exclude	Exclude	Exclude	Exclude	Include	Include	Exclude
Arterial hypertension	Exclude	Exclude	Exclude	Exclude	Exclude	Include	Exclude	Exclude	Exclude	Exclude	Exclude	Exclude	Exclude	Exclude
Baseline oxygen requirement	Exclude	Exclude	Include	Exclude	Exclude	Include	Exclude	Exclude	Exclude	Exclude	Exclude	Include	Include	Include
Diastolic dysfunction	Exclude	Exclude	Exclude	Include	Exclude	Include	Exclude	Exclude	Exclude	Exclude	Exclude	Include	Exclude	Exclude
Valve regurgitation	Exclude	Exclude	Exclude	Exclude	Exclude	Include	Exclude	Exclude	Exclude	Include	Exclude	Include	Exclude	Include
Aortitis	Exclude	Exclude	Include	Exclude	Exclude	Include	Exclude	Exclude	Exclude	Exclude	Exclude	Exclude	Exclude	Exclude
Cardiogenic shock not due to sepsis	Exclude	Exclude	Include	Exclude	Exclude	Exclude	Exclude	Exclude	Exclude	Exclude	Exclude	Exclude	Exclude	Exclude
QT prolongation	Exclude	Exclude	Exclude	Include	Exclude	Exclude	Exclude	Exclude	Exclude	Exclude	Exclude	Exclude	Exclude	Exclude
**PERIPHERAL VASCULAR**														
Significant tissue loss ever (e.g. loss of digit or limb)	Include	Include	Include	Include	Include	Include	Exclude	Include	Include	Include	Include	Include	Include	Include
Claudication	Include	Include	Include	Include	Include	Include	Exclude	Include	Include	Include	Exclude	Include	Include	Include
Intracardiac thrombus	Exclude	Exclude	Include	Include	Exclude	Include	Exclude	Exclude	Include	Exclude	Exclude	Exclude	Exclude	Exclude
Minor tissue loss (pulp space)	Exclude	Include	Include	Include	Exclude	Include	Exclude	Exclude	Include	Exclude	Exclude	Include	Exclude	Include
Renal artery stenosis	Exclude	Include	Include	Include	Exclude	Include	Exclude	Exclude	Include	Exclude	Exclude	Include	Exclude	Include
Severe Raynaud’s	Exclude	Exclude	Include	Exclude	Exclude	Include	Exclude	Exclude	Exclude	Exclude	Exclude	Exclude	Exclude	Exclude
Thrombosis any (arterial or venous)	Exclude	Include	Include	Include	Exclude	Include	Exclude	Include	Exclude	Exclude	Exclude	Include	Exclude	Include
Venous thrombosis or venous stasis	Include	Include	Exclude	Exclude	Exclude	Exclude	Exclude	Exclude	Exclude	Exclude	Exclude	Include	Exclude	Include

Items	Inc/Excl	Inc/Excl	Inc/Excl	Inc/Excl	Inc/Excl	Inc/Excl	Inc/Excl	Inc/Excl	Inc/Excl	Inc/Excl	Inc/Excl	Inc/Excl	Inc/Excl	Inc/Excl
**PULMONARY**														
Pulmonary fibrosis	Include	Include	Include	Include	Include	Include	Exclude	Include	Exclude	Include	Include	Include	Exclude	Include
Pulmonary hypertension	Include	Exclude	Exclude	Include	Include	Exclude	Include	Exclude	Exclude	Include	Include	Include	Exclude	Include
Pleural fibrosis, pleurodesis or pleurectomy for refractory effusions	Include	Include	Include	Include	Include	Include	Include	Include	Include	Exclude	Include	Include	Exclude	Include
Pulmonary infarction or resection not for malignancy	Include	Include	Include	Include	Include	Include	Exclude	Exclude	Include	Include	Include	Include	Exclude	Include
Shrinking lung	Include	Include	Include	Include	Include	Include	Exclude	Exclude	Include	Include	Include	Include	Exclude	Exclude
Chronic diffuse alveolar haemorrhage	Exclude	Exclude	Include	Include	Exclude	Include	Exclude	Exclude	Include	Exclude	Include	Include	Exclude	Exclude
Lobectomy	Exclude	Exclude	Exclude	Include	Exclude	Include	Exclude	Exclude	Exclude	Exclude	Exclude	Include	Exclude	Include
Persistent decreased exercise tolerance/ diffusing capacity for carbon monoxide	Exclude	Include	Include	Exclude	Exclude	Include	Exclude	Exclude	Include	Exclude	Include	Include	Include	Include
Respiratory insufficiency	Exclude	Exclude	Include	Include	Exclude	Exclude	Include	Exclude	Exclude	Exclude	Include	Include	Exclude	Exclude
Restrictive lung disease (based on pulmonary function test abnormalities)	Exclude	Include	Exclude	Exclude	Exclude	Include	Exclude	Exclude	Exclude	Include	Exclude	Include	Exclude	Exclude
Diminished lung function (decreased DLCO, FEV1, FVC)	Exclude	Include	Exclude	Exclude	Exclude	Include	Exclude	Exclude	Include	Exclude	Exclude	Exclude	Exclude	Exclude
Bronchiectasis	Include	Include	Include	Exclude	Exclude	Exclude	Exclude	Exclude	Include	Exclude	Exclude	Include	Exclude	Exclude
Obstructive airway disease	Exclude	Exclude	Exclude	Exclude	Exclude	Exclude	Exclude	Exclude	Exclude	Exclude	Exclude	Exclude	Exclude	Exclude
Obstructive sleep apnoea	Exclude	Exclude	Exclude	Exclude	Exclude	Exclude	Exclude	Exclude	Exclude	Exclude	Exclude	Exclude	Exclude	Exclude

Items	Inc/Excl	Inc/Excl	Inc/Excl	Inc/Excl	Inc/Excl	Inc/Excl	Inc/Excl	Inc/Excl	Inc/Excl	Inc/Excl	Inc/Excl	Inc/Excl	Inc/Excl	Inc/Excl
**MUSCULOSKELETAL**														
Avascular necrosis / Legg-Calve-Perthes disease / Perthes disease	Include	Include	Include	Include	Include	Include	Include	Include	Include	Include	Exclude	Include	Include	Include
Osteoporosis with fracture	Include	Include	Include	Include	Include	Include	Include	Include	Include	Include	Include	Include	Exclude	Include
Erosive arthropathy	Include	Include	Include	Include	Include	Include	Exclude	Exclude	Include	Include	Include	Include	Include	Include
Fragility fractures	Exclude	Exclude	Exclude	Include	Exclude	Include	Exclude	Exclude	Exclude	Include	Include	Exclude	Exclude	Include
Jaccoud's arthropathy	Exclude	Include	Include	Include	Exclude	Include	Include	Include	Include	Include	Include	Include	Include	Exclude
Joint replacement	Include	Include	Include	Include	Exclude	Include	Include	Exclude	Include	Include	Include	Include	Include	Include
Atrophy or weakness	Include	Include	Include	Exclude	Exclude	Exclude	Exclude	Exclude	Include	Exclude	Include	Exclude	Exclude	Exclude
Muscle atrophy	Exclude	Exclude	Exclude	Include	Exclude	Include	Exclude	Exclude	Exclude	Exclude	Exclude	Include	Exclude	Exclude
Osteoporosis without fracture	Exclude	Exclude	Exclude	Include	Exclude	Include	Include	Exclude	Include	Exclude	Exclude	Include	Include	Include
Tendon rupture	Include	Include	Include	Include	Exclude	Include	Include	Include	Include	Include	Include	Include	Exclude	Include
Osteomyelitis	Include	Exclude	Include	Include	Exclude	Exclude	Exclude	Exclude	Include	Exclude	Include	Include	Exclude	Exclude
Sarcopenia	Exclude	Include	Exclude	Exclude	Exclude	Include	Include	Exclude	Exclude	Exclude	Include	Exclude	Exclude	Exclude
Frailty	Exclude	Include	Exclude	Exclude	Exclude	Include	Exclude	Exclude	Exclude	Exclude	Exclude	Exclude	Exclude	Exclude
Osteomalacia	Exclude	Exclude	Exclude	Exclude	Exclude	Exclude	Exclude	Exclude	Exclude	Exclude	Exclude	Exclude	Exclude	Exclude
Fibromyalgia	Exclude	Exclude	Exclude	Exclude	Exclude	Exclude	Exclude	Exclude	Exclude	Exclude	Exclude	Exclude	Exclude	Exclude
Osteosclerosis	Exclude	Exclude	Exclude	Exclude	Exclude	Exclude	Exclude	Exclude	Exclude	Exclude	Exclude	Exclude	Exclude	Exclude
Septic arthritis	Exclude	Exclude	Exclude	Exclude	Exclude	Exclude	Exclude	Exclude	Exclude	Exclude	Exclude	Exclude	Exclude	Exclude
Chronic gout with erosion and tophi	Exclude	Exclude	Exclude	Exclude	Exclude	Exclude	Exclude	Exclude	Exclude	Exclude	Exclude	Exclude	Exclude	Exclude
Inflammatory joint pain	Exclude	Exclude	Exclude	Exclude	Exclude	Exclude	Exclude	Exclude	Exclude	Exclude	Exclude	Exclude	Exclude	Exclude

Items	Inc/Excl	Inc/Excl	Inc/Excl	Inc/Excl	Inc/Excl	Inc/Excl	Inc/Excl	Inc/Excl	Inc/Excl	Inc/Excl	Inc/Excl	Inc/Excl	Inc/Excl	Inc/Excl
**SKIN/MUCOCUTANEOUS**														
Scarring chronic alopecia	Include	Include	Include	Include	Include	Include	Exclude	Include	Include	Include	Include	Include	Include	Include
Scarring (cutaneous)	Exclude	Include	Include	Include	Exclude	Include	Include	Include	Include	Include	Include	Include	Include	Include
Skin ulceration (excluding thrombosis) for > 6 months	Exclude	Include	Include	Include	Include	Include	Include	Include	Exclude	Include	Include	Include	Exclude	Include
Chronic discoid or subacute lesions	Exclude	Include	Include	Include	Exclude	Include	Exclude	Exclude	Exclude	Include	Exclude	Include	Exclude	Include
Cicatricial skin atrophy	Exclude	Include	Exclude	Exclude	Exclude	Include	Exclude	Exclude	Exclude	Include	Include	Include	Include	Include
Dyspigmentation (hyperpigmentation or hypopigmentation)	Include	Include	Include	Include	Include	Include	Exclude	Exclude	Exclude	Exclude	Exclude	Include	Exclude	Exclude
Post venous thrombosis complications	Exclude	Exclude	Exclude	Exclude	Include	Include	Exclude	Exclude	exclude	Include	Exclude	Include	Include	Include
Calcinosis	Exclude	Exclude	Include	Exclude	Exclude	Include	Exclude	Exclude	Include	Exclude	Exclude	Include	Exclude	Exclude
Extensive striae	Exclude	Include	Include	Include	Include	Include	Exclude	Exclude	Include	Include	Exclude	Include	Exclude	Include
Panniculitis	Exclude	Include	Exclude	Include	Exclude	Include	Include	Exclude	Exclude	Exclude	Exclude	Include	Exclude	Exclude
Alopecia without scarring	Include	Exclude	Include	Exclude	Exclude	Include	Exclude	Exclude	Exclude	Exclude	Exclude	Exclude	Exclude	Exclude
Hirsutism	Exclude	Exclude	Include	Exclude	Exclude	Exclude	Exclude	Exclude	Include	Exclude	Exclude	Exclude	Exclude	Exclude
Lipodystrophy	Exclude	Exclude	Include	Include	Exclude	Include	Exclude	Exclude	Include	Exclude	Exclude	Include	Exclude	Exclude
Nasal septum perforation	Exclude	Exclude	Include	Include	Exclude	Include	Exclude	Exclude	Include	Exclude	Exclude	Include	Exclude	Exclude
Periodontal disease with tooth loss	Include	Exclude	Exclude	Exclude	Exclude	Include	Exclude	Exclude	Include	Exclude	Exclude	Include	Include	Exclude
Persistent malar rash	Exclude	Exclude	Exclude	Exclude	Exclude	Include	Exclude	Exclude	Exclude	Exclude	Exclude	Exclude	Exclude	Exclude
Severe livedo reticularis	Exclude	Include	Exclude	Exclude	Exclude	Include	Exclude	Exclude	Include	Exclude	Exclude	Exclude	Exclude	Exclude
Severe photosensitivity limiting activities of daily living	Exclude	Exclude	Exclude	Exclude	Exclude	Include	Exclude	Include	Exclude	Exclude	Exclude	Exclude	Exclude	Exclude
Sjogrens syndrome	Include	Exclude	Exclude	Exclude	Include	Exclude	Exclude	Exclude	Exclude	Exclude	Exclude	Include	Exclude	Exclude
Telangiectasia	Exclude	Include	Exclude	Exclude	Exclude	Include	Exclude	Include	Exclude	Exclude	Exclude	Exclude	Exclude	Exclude
Xerostomia	Exclude	Exclude	Exclude	Exclude	Exclude	Include	Exclude	Exclude	Exclude	Exclude	Exclude	Exclude	Exclude	Exclude
Easy bruisability	Exclude	Exclude	Exclude	Exclude	Exclude	Exclude	Exclude	Exclude	Exclude	Exclude	Exclude	Exclude	Exclude	Exclude
Onychodystrophy	Exclude	Exclude	Exclude	Exclude	Exclude	Exclude	Exclude	Exclude	Exclude	Exclude	Exclude	Exclude	Exclude	Exclude

Items	Inc/Excl	Inc/Excl	Inc/Excl	Inc/Excl	Inc/Excl	Inc/Excl	Inc/Excl	Inc/Excl	Inc/Excl	Inc/Excl	Inc/Excl	Inc/Excl	Inc/Excl	Inc/Excl
**OTHERS**														
Malignancy	Include	Include	Include	Include	Include	Include	Include	Include	Include	Include	Include	Include	Include	Exclude
Organ transplantation (non-kidney)	Include	Include	Include	Include	Include	Include	Exclude	Exclude	Include	Include	Include	Include	Include	Include
Treatment related damage	Exclude	Exclude	Exclude	Exclude	Include	Include	Include	Exclude	Exclude	Exclude	Exclude	Exclude	Exclude	Include
Persistent hypogammaglobulinemia	Exclude	Include	Include	Include	Exclude	Include	Exclude	Exclude	Include	Include	Exclude	Include	Include	Exclude
Severe or recurrent infections	Include	Include	Include	Exclude	Exclude	Exclude	Include	Exclude	Exclude	Exclude	Include	Exclude	Exclude	Exclude
Carcinoma in situ (any site)	Exclude	Exclude	Exclude	Include	Exclude	Exclude	Exclude	Exclude	Include	Exclude	Exclude	Exclude	Include	Exclude
Work disability	Exclude	Exclude	Exclude	Exclude	Exclude	Include	Exclude	Exclude	Exclude	Exclude	Exclude	Exclude	Exclude	Exclude
Social isolation	Exclude	Exclude	Exclude	Exclude	Exclude	Exclude	Exclude	Exclude	Exclude	Exclude	Exclude	Exclude	Exclude	Exclude
Poverty	Exclude	Exclude	Exclude	Exclude	Exclude	Exclude	Exclude	Exclude	Include	Exclude	Exclude	Exclude	Exclude	Exclude

*Top-row colors identify individual Item Reduction Committee members. Cell colors denote voting outcomes: blue = include; yellow = possible exclude (e.g. due to overlap); red = strict exclude. Items receiving strict exclude votes from ≥5 Committee members were removed

Supplementary Table 4. Excluded items and exclusion reasons by the Clinical Domain Groups

Items	Exclusion reason
**NEUROPSYCHIATRIC**	
Peripheral neuropathy	Reversible
Movement disorders	Reversible
Psychosis	Reversible
**GROWTH AND DEVELOPMENT**	
Delayed puberty/Disordered puberty sequences, e.g. reaching menarche without/with minimal/ less than expected growth spurt, breast or pubic hair development	Overlapping, rare
**HEMATOLOGIC**	
Splenectomy	Overlapping
Bone marrow failure	Reversible
Non-Hodgkin's lymphoma	Overlapping
Spleen infarction	Overlapping
Treatment-related myelodysplastic syndrome	Reversible, rare
**GASTROINTESTINAL**	
Bowel stricture	Rare
Chronic liver disease and cirrhosis	Rare
Chronic Peritonitis	Rare
End stage liver disease	Rare
Pancreatic insufficiency or need to use pancreatic enzyme replacement	Rare
**PULMONARY**	
Pleural fibrosis, pleurodesis or pleurectomy for refractory effusions	Rare
Shrinking lung	Rare
**SKIN/MUCOCUTANEOUS**	
Skin ulceration (excluding thrombosis)	Rare
Extensive striae	Overlapping
