# Supplementary material for: The lupus damage index revision program: Results from the item generation and reduction phases
Source: Lupus Sci Med. 2026 Aug 12;13(2):e002282. doi: 10.1136/lupus-2026-002282 (PMC13475098; doi:10.1136/lupus-2026-002282)

Figure 1 a and b: Anatomical boundaries of a) the scalp and b) the four quadrants of the scalp using the Cutaneous Lupus Erythematosus Disease Area and Severity Index (CLASI) boundaries[74]

1a

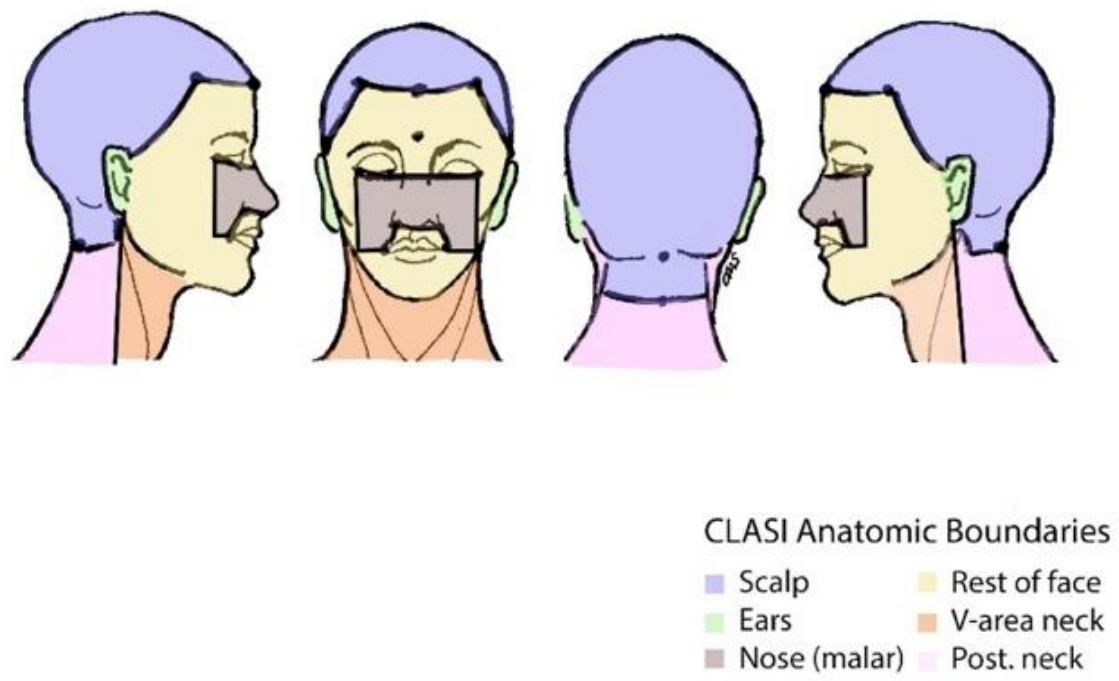

1b

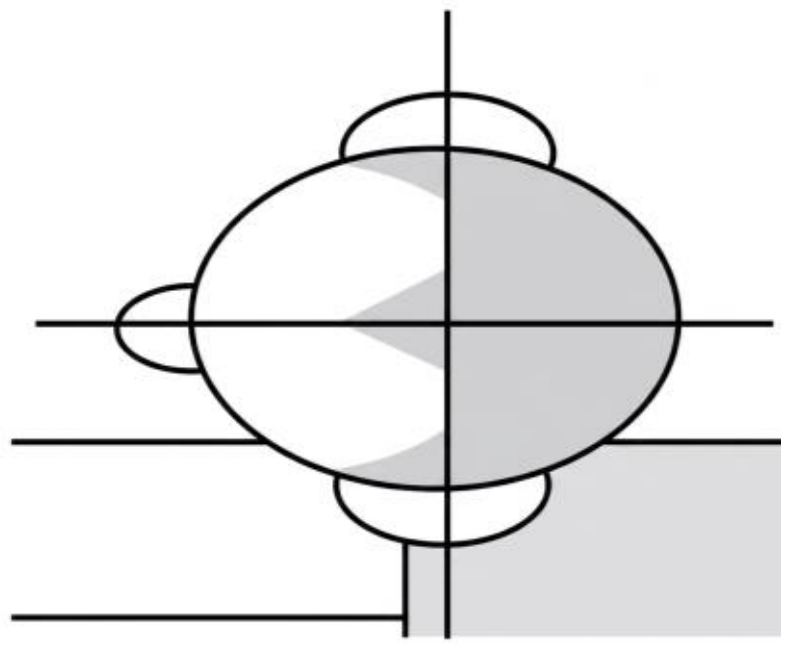

Supplement: Supplementary file 3 [file lupus-13-2-s003.pdf]
