# Supplementary material for: The lupus damage index revision program: Results from the item generation and reduction phases
Source: Lupus Sci Med. 2026 Aug 12;13(2):e002282. doi: 10.1136/lupus-2026-002282 (PMC13475098; doi:10.1136/lupus-2026-002282)
Supplement: Supplementary file 4 [file lupus-13-2-s004.pdf]

Figure 2. Number of candidate items identified during item generation and item reduction phases of revised SDI development

Item generation: Literature review and first Delphi round

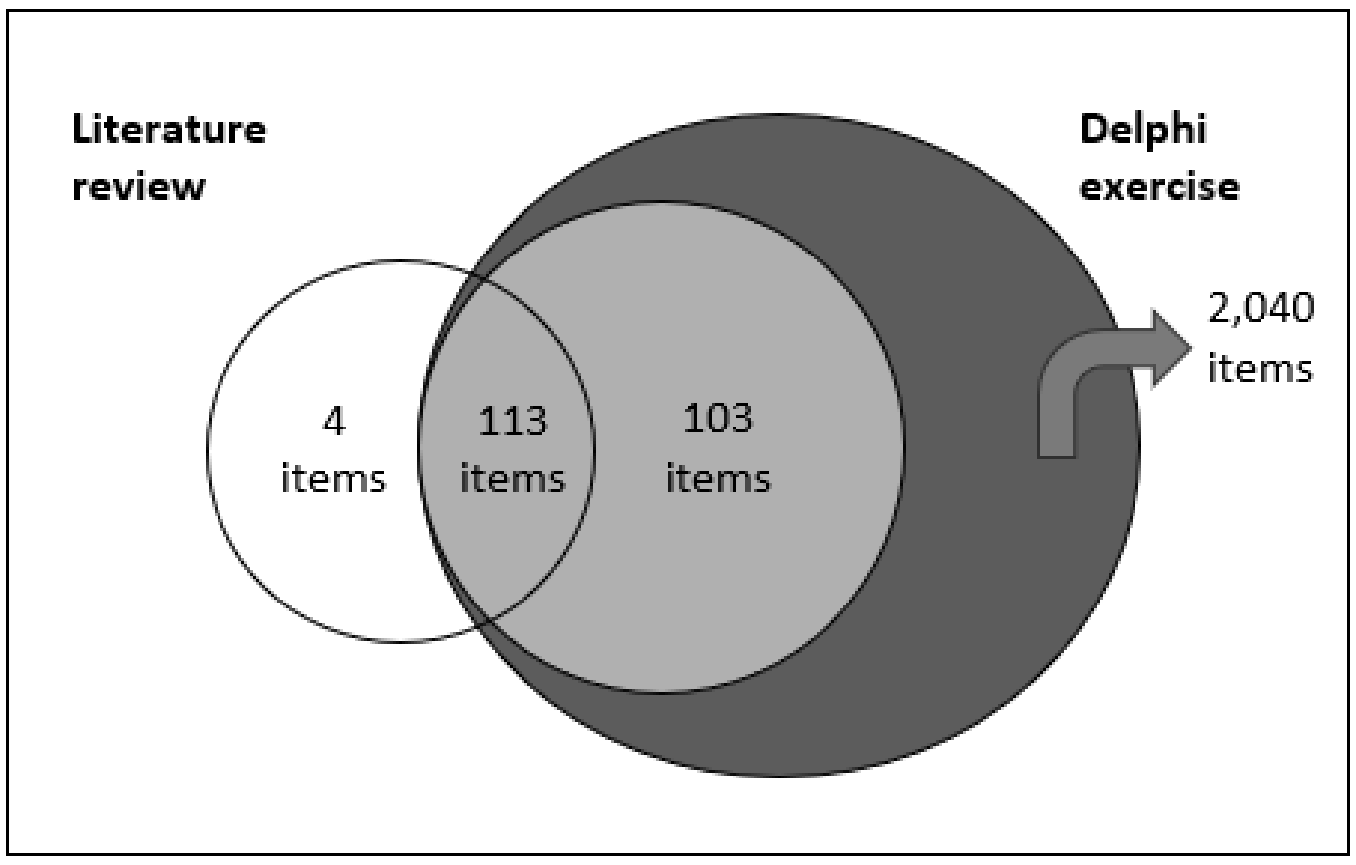

220 candidate items

↓

Item generation: Second Delphi round
(Additional items coming from second Delphi round)

226 candidate items

↓

Item reduction: Third Delphi Round

190 candidate items

↓

Item reduction: Item Reduction Committee

64 candidate items

↓

Item reduction: Clinical Domain Groups

39 candidate items

* 2,040 initial ('raw') items were removed following clustering of overlapping items and harmonization of synonymous terms.
